# Beyond Genes: Inclusion of Alternative Splicing and Alternative Polyadenylation to Assess the Genetic Architecture of Predisposition to Voluntary Alcohol Consumption in Brain of the HXB/BXH Recombinant Inbred Rat Panel

**DOI:** 10.3389/fgene.2022.821026

**Published:** 2022-03-15

**Authors:** Ryan Lusk, Paula L. Hoffman, Spencer Mahaffey, Samuel Rosean, Harry Smith, Jan Silhavy, Michal Pravenec, Boris Tabakoff, Laura M. Saba

**Affiliations:** ^1^ Department of Pharmaceutical Sciences, University of Colorado Anschutz Medical Campus, Aurora, CO, United States; ^2^ Department of Pharmacology, University of Colorado Anschutz Medical Campus, Aurora, CO, United States; ^3^ Institute of Physiology of the Czech Academy of Sciences, Prague, Czechia

**Keywords:** HXB/BXH recombinant inbred panel, voluntary alcohol consumption, transcriptome, weighted gene co expression network analysis, alternative splicing, alternative polyadenylation, RNA-seq—RNA sequencing, isoform

## Abstract

Post transcriptional modifications of RNA are powerful mechanisms by which eukaryotes expand their genetic diversity. For instance, researchers estimate that most transcripts in humans undergo alternative splicing and alternative polyadenylation. These splicing events produce distinct RNA molecules, which in turn yield distinct protein isoforms and/or influence RNA stability, translation, nuclear export, and RNA/protein cellular localization. Due to their pervasiveness and impact, we hypothesized that alternative splicing and alternative polyadenylation in brain can contribute to a predisposition for voluntary alcohol consumption. Using the HXB/BXH recombinant inbred rat panel (a subset of the Hybrid Rat Diversity Panel), we generated over one terabyte of brain RNA sequencing data (total RNA) and identified novel splice variants (*via* StringTie) and alternative polyadenylation sites (*via* aptardi) to determine the transcriptional landscape in the brains of these animals. After establishing an analysis pipeline to ascertain high quality transcripts, we quantitated transcripts and integrated genotype data to identify candidate transcript coexpression networks and individual candidate transcripts associated with predisposition to voluntary alcohol consumption in the two-bottle choice paradigm. For genes that were previously associated with this trait (e.g., *Lrap*, *Ift81*, and *P2rx4*) (Saba et al., Febs. J., 282, 3556–3578, Saba et al., Genes. Brain. Behav., 20, e12698), we were able to distinguish between transcript variants to provide further information about the specific isoforms related to the trait. We also identified additional candidate transcripts associated with the trait of voluntary alcohol consumption (i.e., isoforms of *Mapkapk5*, *Aldh1a7*, and *Map3k7*). Consistent with our previous work, our results indicate that transcripts and networks related to inflammation and the immune system in brain can be linked to voluntary alcohol consumption. Overall, we have established a pipeline for including the quantitation of alternative splicing and alternative polyadenylation variants in the transcriptome in the analysis of the relationship between the transcriptome and complex traits.

## 1 Introduction

Post transcriptional phenomena are powerful mechanisms by which eukaryotes expand their genetic diversity. For instance, researchers estimate that 95–100% of multi-exon genes in human can undergo alternative splicing (Pan et al., 2008; Wang et al., 2008; Nilsen and Graveley, 2010). Likewise, an estimated 70% or more mammalian genes have multiple polyadenylation sites (Derti et al., 2012; Hoque et al., 2013) and can therefore express alternative polyadenylation isoforms. Biologically, alternative splicing of exons can lead to diverse functions (Garcia-Blanco et al., 2004) by changing the protein encoded by the mRNA (Kelemen et al., 2013). In contrast, the vast majority of alternative polyadenylation occurs in the 3′ untranslated region (3′ UTR) (Tian and Manley, 2017) and thus generates identical proteins. However, alternative polyadenylation profoundly impacts the mRNA by modifying its stability, translocation, nuclear export, and cellular localization, as well as the localization of the encoded protein (Yeh and Yong, 2016; Tian and Manley, 2017). Alternative polyadenylation most often exerts its effects through gain or loss of microRNA binding sites in the 3′ UTR; more than 50% of conserved microRNA binding sites reside downstream of the most proximal polyadenylation site in mammalian genes (Ren et al., 2020).

Alternative splicing and alternative polyadenylation have increasingly been associated with disease. For example, alternative splicing has been recognized as a genetic modifier of disease phenotype (Garcia-Blanco et al., 2004) and susceptibility to disease (Wang and Cooper, 2007). Notably, alternative splicing has also been shown to impact the phenotypic variation of diseases, or in other words impact quantitative (complex) traits, via changes in the relative expression levels among different mRNA isoforms produced from the same gene (Nissim-Rafinia and Kerem, 2002). One example of the latter is the tau protein; exon 10 of this gene can be included or skipped, and dysregulation of the ratio between these two alternative splicing isoforms can lead to the development of inherited frontotemporal dementia and parkinsonism (Hong et al., 1998; Hutton et al., 1998; Spillantini et al., 1998). Furthermore, several other alternative splicing events have been linked to neurological diseases (Dredge et al., 2001).

Although alternative polyadenylation is a relatively new research area, it too has been associated with biological processes such as the innate antiviral immune response, cancer initiation and prognosis, and development of drug resistance (Ren et al., 2020). Alternative polyadenylation also affects brain function. For instance, the serotonin transporter has two 3′ UTR alternative polyadenylation isoforms, the longer of which is implicated in anxiolytic effects (Hartley et al., 2012; Yoon et al., 2013). The long 3′ UTR isoform of the *alpha-synuclein* gene is associated with Parkinson’s disease pathology (Rhinn et al., 2012), and a shift to increased usage of the long 3′ UTR version of this gene has been shown in response to elevated cytoplasmic dopamine levels and other conditions causing oxidative stress (Miura et al., 2014). Only the long 3′ UTR version was found to mediate translational upregulation of the BDNF protein, and modulation of activity-dependent neuronal signaling is thought to predispose individuals to many psychiatric disorders (Lau et al., 2010). Overall, differences in expression of alternative polyadenylation transcripts have been implicated in disease (Yoon et al., 2012) and alternative polyadenylation is increasingly being acknowledged as a risk factor for several complex traits (Manning and Cooper, 2017).

Importantly, these post-transcriptional modifications are dynamic. For example, alternative splicing patterns constantly change under specific physiological conditions (Kelemen et al., 2013) and are modulated according to cell type, developmental stage, sex, or in response to stimuli (Faustino and Cooper, 2003). Additionally, *in vivo* expression of alternative polyadenylation isoforms varies based on a myriad of factors such as tissue, physiological, and disease states (Di Giammartino et al., 2011; Sanfilippo et al., 2017) and displays tissue specificity (Beaudoing and Gautheret, 2001; Zhang et al., 2005). Of note, the brain expresses the greatest mRNA diversity compared to other tissues due to alternative splicing and alternative polyadenylation (MacDonald, 2019), and broad neural 3′ UTR lengthening is ubiquitous in the adult mammalian brain (Miura et al., 2014).

However, reference annotation (especially for non-human organisms such as the rat) often lacks annotation of alternative splicing and alternative polyadenylation transcripts. Likewise, reference annotation, in general, represents data pooled from multiple tissues, experiments, etc., and thus may not accurately represent the transcriptome for a given organ or condition.

The objective in our present study was twofold: 1) characterize the alternative splicing and alternative polyadenylation landscape in brain of the HXB/BXH recombinant inbred (RI) rat panel, and 2) identify candidate RNA transcripts associated with the complex trait of voluntary alcohol (i.e., ethanol) consumption using both a network and individual transcript approach to better understand biological mechanisms related to why some animals drink more than others. Throughout this paper, we use the term “transcript” to refer to the sequence of a processed mature RNA and the term “gene” as the DNA locus that codes for a particular transcript or set of transcripts. In this context a single gene can produce many distinct transcripts through alternative splicing and alternative transcription start and stop sites (e.g., alternative polyadenylation).

Alcohol use disorder (AUD) and its endophenotypes, including alcohol consumption [an etiologic essential to the development of AUD (Grant et al., 2009; Dawson and Grant, 2011)], have a noteworthy genetic component (Verhulst et al., 2015). Yet much of the genetic architecture of alcohol related phenotypes remains unclear. Traditionally, scientists investigated the role of alternative splicing and alternative polyadenylation on alcohol related phenotypes by interrogating individual candidate genes. For example, studies on N-methyl-d-aspartate (NMDA) receptor found greater basal expression of a subunit splicing variant in alcohol non-preferring rats compared to alcohol preferring rats in the hippocampus (Winkler et al., 1999). Besides NMDA, alternative splicing of DRD2, GABA_A_ receptor, ion channels, voltage-gated Ca^2+^ channels, and neurexin-3, among others, have been linked to various alcohol related phenotypes (Sasabe and Ishiura, 2010). Recent advances in next generation sequencing and have allowed researchers to characterize alternative splicing and alternative polyadenylation across the entire transcriptome to increase our understanding of their roles. For instance, alcohol use disorder was associated with global changes in splicing (i.e., alternative splicing and alternative polyadenylation) in human brains and, furthermore, may be controlled by long non-coding RNAs that act as master regulators of splicing (Van Booven et al., 2021). Changes in expression of splice variants were linked to alcohol related associated learning in the brains of fruit flies, and knockdown of splicesome-associated proteins prevent the formation of alcohol memories (Petruccelli et al., 2020). In addition, significant changes in alternative splicing were observed between alcohol- and control-exposed human fetal cortical samples (Kawasawa et al., 2017).

To accomplish our first goal, we utilized an extensive RNA expression database from whole brain samples of the HXB/BXH RI panel that consisted of over one terabyte of RNA sequencing (RNA-Seq) data. We then applied two computational methods, StringTie (Pertea et al., 2015) and aptardi (Lusk et al., 2021), to characterize *in vivo* expression of alternative splice variants and alternative polyadenylation in the brains of these animals. We furthermore developed a transcriptome reconstruction pipeline that integrates these tools (Pertea et al., 2015; Lusk et al., 2021) and also filters transcripts to eliminate potential false positives yielding high-quality transcriptome information.

To accomplish our second goal, we focused on examining the role of quantitative RNA expression levels as possible mediators of genetic determinants of alcohol consumption. We integrated RNA expression data with phenotypic data to identify candidate coexpression modules and individual candidate transcripts that are associated with a rat’s predisposition to voluntarily consume more or less alcohol than other rats, i.e., RNA expression data were obtained from animals which were not tested for alcohol preference and the behavioral data was obtained from genetically identical rats (i.e., rats from the same inbred strain), as done in our prior work (Tabakoff et al., 2009; Saba et al., 2015; Harrall et al., 2016; Pravenec et al., 2018). Particularly, we had previously used whole brain RNA expression data and the identical phenotype of voluntary alcohol consumption to identify candidate gene coexpression modules associated with the alcohol drinking phenotype (Saba et al., 2015). However, our earlier study utilized expression data obtained from microarrays and analysis was limited to genes and transcripts that were unambiguously probed by the microarray. In the current study, we can quantify all RNA-Seq amenable transcripts expressed in brain and identify specific transcripts associated with the alcohol drinking trait.

## 2 Materials and Methods

### 2.1 Animals

The HXB/BXH RI rat panel, a subset of the Hybrid Rat Diversity Panel (HRDP) (Tabakoff et al., 2019), was used in this study. This RI panel consists of 30 inbred strains derived from the congenic Brown Norway strain with polydactyly-luxate syndrome (BN-Lx/Cub) and the Wistar origin spontaneously hypertensive rat strain (SHR/OlaIpcv) using gender reciprocal crossing and more than 80 generations of brother sister mating after the F2 generation (Pravenec et al., 1989). This subset of the HRDP was used because phenotype data is publicly available on alcohol consumption (Tabakoff et al., 2009) and other alcohol-related phenotypes (Lusk et al., 2018).

### 2.2 Voluntary Alcohol Consumption in the HXB/BXH Recombinant Inbred Rat Panel

Voluntary alcohol consumption was measured using a two-bottle choice paradigm in 23 HXB/BXH RI strains and the two progenitor strains of the RI panel. The alcohol consumption phenotype was measured in male rats that were 70–100 days old at the beginning of the study. The two-bottle choice paradigm and voluntary alcohol consumption measurements during week two of the paradigm are described in our previous study (Tabakoff et al., 2009) and constituted the voluntary alcohol consumption phenotype. We have used this phenotype in previous genetics studies (Tabakoff et al., 2009; Vanderlinden et al., 2014; Saba et al., 2015, Saba et al., 2020) and established its heritability (R^2^ = 0.39) (Tabakoff et al., 2009), making it amenable to genomics studies.

### 2.3 Whole Brain RNA Sequencing

The University of Colorado Anschutz Medical Campus received shipments of brain tissue from male rats (∼70–90 days old) stored in liquid nitrogen from Dr. Michal Pravenec at the Institute of Physiology of the Czech Academy of Sciences. These animals were maintained in accordance with the Animal Protection Law of the Czech Republic and were approved by the Ethics Committee of the Institute of Physiology, Czech Academy of Sciences, Prague. A total 90 HXB/BXH brains from individual rats (three each from 30 HXB/BXH RI strains) were received, as well as three brains from the SHR/OlaIpcv progenitor strain (93 brains total). The SHR/OlaIpcv brains were used as loading controls, and technical replicate(s), i.e., multiple sequencing libraries, were generated and assayed in each sequencing batch to assess reproducibility of the RNA-Seq results. Including these technical replicates in the SHR/OlaIpcv strain, 100 sequencing libraries were generated from 93 rat brains.

Total RNA (>200 nucleotides) was extracted from whole brain using the RNeasy Plus Universal Midi Kit (Qiagen, Valencia, CA, United States) and cleaned using the RNeasy Mini Kit (Qiagen, Valencia, CA, United States). Four μL 1:100 dilution of either ERCC Spike-In Mix 1 or Mix 2 (ThermoFisher Scientific, Wilmington, DE, United States) was added to each RNA sample. The Illumina TruSeq Stranded RNA Sample Preparation kit (Illumina, San Diego, CA, United States) was used to construct sequencing libraries, which includes a ribosomal RNA depletion step that uses the Ribo-Zero rRNA reduction chemistry. Sequencing library quality was evaluated using an Agilent Technologies Bioanalyzer 2100 (Agilent Technologies, Santa Clara, CA, United States). Samples were sequenced in eight batches on an Illumina HiSeq2500 or HiSeq4500 (Illumina, San Diego, CA, United States) in High Output mode to generate 2 × 100 or 2 × 150 paired end reads.

### 2.4 Brain Specific Transcriptome Reconstruction and Quantitation Using Whole Brain RNA Sequencing

Brain-specific transcriptome information for the HXB/BXH RI panel used in this study was generated by incorporating RNA expression data (in the form of short read RNA-Seq), DNA sequence information, and computational methods, namely StringTie (Pertea et al., 2015) and aptardi (Lusk et al., 2021), to annotate expressed alternative splicing and alternative polyadenylation transcripts, respectively. In particular, StringTie utilizes RNA-Seq data to reconstruct the transcriptome and identify expressed transcripts not present in the Ensembl reference annotation. However, StringTie only identifies single polyadenylation sites per transcript, i.e., it cannot identify alternative polyadenylation transcript isoforms (Faustino and Cooper, 2003). In contrast, aptardi is designed to evaluate a set of input transcripts and annotate any alternative polyadenylation sites based on the corresponding RNA-Seq data and surrounding DNA sequence to aid in its identification. Therefore, applying these computational methods enables the characterization of the expressed transcriptome to identify alternative splicing and alternative polyadenylation transcripts not in the Ensembl reference annotation but supported by RNA-Seq expression data specific to the sample(s). The transcriptome was then quantitated at the individual transcript level to enable downstream quantitative analyses for evaluating the role of transcripts in predisposition to voluntary alcohol consumption. An outline of the transcriptome reconstruction and quantitation steps are presented in Supplementary Figures S1, S2.

#### 2.4.1 Read Processing for Quality

Initially, adapter sequences and low quality base calls were eliminated from raw reads using cutadapt (v.1.9.1) (Martin, 2011). Reads were removed from further analysis if they aligned to rRNA from the RepeatMasker database (Smit et al., 1996) (accessed through the UCSC Genome Browser; https://genome.ucsc.edu/) (Kent et al., 2002). This alignment was done using Bowtie 2 (v.2.3.4.3) (Langmead and Salzberg, 2012).‬‬‬‬‬‬‬‬‬

#### 2.4.2 Evaluation of Unannotated Genes and Unannotated Splicing—StringTie Transcriptome Reconstruction

Individual RNA-Seq libraries were aligned to their strain-specific genomes using HISAT2 (v.2.1.0) (Kim et al., 2015), and then alignments from libraries produced from brains of animals of the same strain were concatenated using SAMtools (v.1.9) (Li et al., 2009) *merge* to generate a single genome alignment per strain. Strain-specific genomes were constructed from the Rat Genome Sequencing Consortium (RGSC) Rnor_6.0/rn6 version of the rat genome (Gibbs et al., 2004) by imputing single nucleotide polymorphism (SNP) information for each strain based on their STAR Consortium genotypes (STAR Consortium, 2008) and DNA sequencing (DNA-Seq) data from male rats of the progenitor strains (Hermsen et al., 2015). StringTie (v.1.3.5) (Pertea et al., 2015) was used to generate *de novo* strain-specific transcriptomes using default settings and included the strain-specific genome alignment and the rat Ensembl reference transcriptome (v.99) (Yates et al., 2020) to guide transcriptome assembly. A combined StringTie transcriptome for the HXB/BXH RI panel was generated using the merge functionality of StringTie by providing all strain-specific transcriptomes and the rat Ensembl reference transcriptome as a guide.

#### 2.4.3 Evaluation of 3′ Termini—Aptardi Transcriptome Reconstruction

The transcriptome was further processed by aptardi (v.1.0.0) to identify alternative polyadenylation sites, i.e., 3′ termini (Lusk et al., 2021). While transcriptome assemblers (e.g., StringTie) that consider expression data can identify study-specific transcripts, they are not designed to identify transcripts that share exon junctions (i.e., splice sites) and only differ at the location of the polyadenylation site (i.e., alternative polyadenylation transcripts). In contrast, aptardi is designed to specifically probe the 3′ ends of transcripts in order to identify multiple polyadenylation sites per transcript. The input transcripts for aptardi analysis were provided by the combined StringTie transcriptome. By considering expression data, aptardi can annotate unique expressed polyadenylation sites. The strain-specific BAM files of all HXB/BXH RI strains (but not the SHR/OlaIpcv BAM file) were merged into a single BAM file using SAMtools merge to generate the aligned RNA expression data for aptardi. Finally, aptardi leverages DNA sequence indicators of polyadenylation to better predict polyadenylation sites. The rat Rnor_6.0/rn6 reference genome accessed via the UCSC Genome Browser (Gibbs et al., 2004; Havlak et al., 2004) was used for genomic sequence information. If more than one polyadenylation site in the 3′ area of a transcript is identified by aptardi, each transcript/polyadenylation site pair is enumerated as a separate transcript by aptardi. All of the input transcript/3′ terminus pairs are also included in the aptardi output, i.e., aptardi only adds transcript/3′terminus pairs.

#### 2.4.4 Detected Above Background Transcriptome Generation and Quantitation

The strength of aptardi is the identification of active polyadenylation sites within a specific genomic region and it does not currently include computational methodology to identify the correct transcript/3′ terminus pair when multiple transcripts have 3′ termini in the region and multiple polyadenylation sites are identified in the region. Instead, it enumerates all transcript/3′ termini pairs. One way to determine the correct pairing of transcript and 3′ terminus in this scenario is to examine the RNA expression levels estimated by RSEM, RNA-Seq by Expectation-Maximization (Li and Dewey, 2011). Correct pairings should have higher expression values than incorrect pairings since RSEM uses an iterative method to “assign” a read to a transcript when the read aligns to multiple transcripts. Therefore, quantitation was used to establish the detected above background (DABG) transcriptome.

Prior to quantitation, libraries with less than 10 million paired end raw reads were removed from all subsequent analyses, resulting in the removal of a single SHR/OlaIpcv library. Also, transcripts not derived from autosomal or sex chromosomes were removed, i.e., transcripts derived from contigs in the current rat genome. Transcripts in the aptardi transcriptome were then quantitated in each of the 90 HXB/BXH RNA-Seq libraries using RSEM (v.1.3.0) (Li and Dewey, 2011). Transcripts with zero estimated read counts in one third or more of these 90 libraries were removed, as well as transcripts that were 200 nucleotides or fewer in length. This high-quality transcriptome was then used to re-quantitate transcripts with RSEM for each of the HXB/BXH libraries as well as the SHR/OlaIpcv libraries. This re-quantitation step was used to allow the reads that originally aligned to a transcript with low expression to be reassigned to a transcript with higher RNA expression levels. Once again, transcripts with zero counts in one third or more of samples were removed to yield the DABG transcriptome and the corresponding estimated read counts for each RNA-Seq library and for each transcript.

Estimated read counts of transcripts in the DABG transcriptome for each RNA-Seq library generated by RSEM were normalized for sequencing depth using upper quartile normalization (Bullard et al., 2010) implemented in the *EDASeq* R package (v.2.22.0) (Risso et al., 2011) followed by a regularized log (rlog) normalization with the *DESeq2* R package (v.1.28.1) (Love et al., 2014). Finally, expression values were adjusted for batch effects using ComBat (Johnson et al., 2007) from *sva* R package (v.3.36.0) (Leek et al., 2012).

### 2.5 Quantitative Trait Loci Analysis

#### 2.5.1 Genetic Markers for Quantitative Trait Loci Analyses

Genetic makers were initially procured from publicly available SNP genotype data for the HXB/BXH RI rats originally published by the STAR Consortium (STAR Consortium, 2008). Probes from the original array were aligned to the Rnor_6.0/rn6 version of the rat genome using BLAT (Kent, 2002). Markers were further processed into unique strain distribution patterns for QTL analyses as detailed in our previous work (Lusk et al., 2018).

#### 2.5.2 Statistical Methods for Quantitative Trait Loci Analyses

Quantitative trait loci (QTL) analysis was performed using marker regression for the behavioral phenotype (pQTL), for module eigengenes (meQTL), and for transcript expression levels (eQTL) to calculate the logarithm of odds (LOD) scores for each SNP as described by Broman and Sen (Broman and Sen, 2009). eQTL and meQTL were only calculated for transcripts/module eigengenes that were associated with alcohol consumption. All empirical genome-wide *p*-values were calculated using 1,000 permutations (Churchill and Doerge, 1994). Both significant (genome-wide *p*-value < 0.05) and suggestive (genome-wide *p*-value < 0.63) (Lander and Kruglyak, 1995) QTL were considered for pQTL analysis (Lander and Kruglyak, 1995; Abiola et al., 2003). For meQTL and eQTL analyses, a stricter genome-wide significant *p*-value of <0.01 was enforced and only the most significant QTL per transcript or module eigengene was considered. The 95% Bayesian credible intervals of significant or suggestive pQTL, significant meQTL, and significant eQTL were estimated as described by Sen and Churchhill (Sen and Churchill, 2001) and all QTL analyses and graphics were generated using the R/qtl package (v.1.47-9) (Broman and Sen, 2009). Strain mean voluntary alcohol consumption values were also used for pQTL analysis. Strain mean transcript normalized expression estimates were used for eQTL analyses, and module eigengene values (which were produced from WGCNA using strain mean transcript normalized expression estimates and thus represent strain means) were used for meQTL analysis.

### 2.6 Heritability of Transcripts

Heritability of transcripts in the DABG transcriptome was estimated as the coefficient of determination (R^2^) from a one-way ANOVA of individual rat expression values using strain as the predictor and transcript normalized expression estimates as the response.

### 2.7 Identification of Candidate Coexpression Networks and Candidate Individual Transcripts Associated With Voluntary Alcohol Consumption

RNA expression data from alcohol naïve rats were used to determine candidate coexpression modules and individual candidate transcripts that are associated with a predisposition to higher or lower levels of voluntary alcohol consumption. Prior to these analyses, additional filtering of the DABG transcriptome was performed to include only transcripts that 1) were the dominant isoforms expressed for a gene (i.e., the three transcripts of a gene with the highest expression based on mean transcripts per million), 2) demonstrated heritability in the HXB/BXH RI panel and thus genetic influence on RNA expression levels, and 3) could be associated with an Ensembl Gene ID (i.e., shared at least one splice junction with an annotated Ensembl transcript) for annotation purposes. Of the 59,751 transcripts in the DABG transcriptome, 37,453 (63%) were kept after removing transcripts that were not within the top three expressed isoforms for a gene. Eliminating transcripts equal to or below the median heritability across all transcripts (heritability <= 0.478), resulted in 20,442 transcripts. Finally, removing transcripts without an associated Ensembl Gene ID produced a final set of 18,543 transcripts (Supplementary Figure S3). The final set of transcripts were derived from 12,609 genes, of which 7,945 (63%), 3,403 (27%), and 1,261 (10%) possessed one, two, or three isoforms, respectively. Of the 18,543 transcripts, 5,427 (29%), 4,932 (27%), and 8,175 (44%) transcripts were identified by aptardi, StringTie, or the Ensembl reference transcriptome, respectively.

#### 2.7.1 Weighted Gene Coexpression Network Analysis

The *WGCNA* R package (v.1.69) (Langfelder and Horvath, 2008) was used to build a transcript coexpression network and to identify coexpression modules within that network from the strain mean normalized expression estimates for individual transcripts. Minimum module size was set to five and the deepSplit parameter was set to four to promote identification of smaller modules, but otherwise default settings were used including a Pearson correlation to determine the initial adjacency matrix. A soft-thresholding index (β) of seven was chosen to approximate a scale-free topology (Zhang and Horvath, 2005) in an unsigned network (Supplementary Figure S4). The module eigengene (first principal component) was used to summarize transcript expression values within a module across strains.

#### 2.7.2 Candidate Coexpression Networks and Individual Candidate Transcripts

Multiple criteria similar to those previously established (Lusk et al., 2018) were used to determine candidate modules and individual candidate transcripts; 1) expression of the transcripts/module is correlated with voluntary alcohol consumption (Spearman correlation coefficient *p*-value < 0.01) (For the module analysis the module eigengene expression values were used for correlation, and for the individual transcripts the strain mean normalized expression estimates were used.), 2) The module eigengene QTL (meQTL; for modules) or expression QTL (eQTL; for individual transcripts) must have genome-wide significance (genome-wide *p*-value < 0.01), and 3) meQTL or eQTL must overlap the significant (genome-wide *p*-value < 0.05) or suggestive (genome-wide *p*-value < 0.63) pQTL where overlap was considered if the SNP with the highest LOD score for the meQTL/eQTL analyses fell within the 95% Bayesian credible interval for the pQTL.

### 2.8 Single Molecule RNA Sequencing (Iso-Seq)

Publicly available single molecule RNA sequencing (Iso-Seq) data from rat brain was used to qualitatively validate splicing and active polyadenylation sites (SRA Accession ID: PRJNA801761). Briefly, total RNA (>200 bases) was extracted from brain samples from a single male rat from the F344/Stm inbred strain and a single male rat from the LE/Stm inbred strain and cleaned using the RNeasy Plus Universal Midi Kit and RNeasy Mini Kit, respectively (Qiagen, Valencia, CA, United States). RNA was transferred to GeneWiz, Inc. (South Plainfield, NJ, United States) for processing on the PacBio Sequel platform using two SMRT cells per sample. PacBio Iso-Seq SMRTbell library preparation followed the manufacturer’s protocol and high and low quality concensus reads (CCS) were transferred to University of Colorado Anschutz Medical Campus for further bioinformatic processing. CCS were aligned to the rat genome (RN6) using minimap2 [v. 2.17 with the flags (-ax splice:hq -uf -secondary = yes -N 15 -K 1G -t 16 -k 12 -w 4); (Li, 2018)]. Alignment files were converted to GTF (gene transfer format) for upload and visualization with the UCSC Genome Browser (https://genome.ucsc.edu/; Kent et al., 2002).

## 3 Results

### 3.1 Whole Brain RNA Sequencing

After processing reads for quality, the number of paired end reads in the 90 HXB/BXH RI panel RNA-Seq libraries ranged from 29 million to 199 million (median number of paired end reads per sample = 71 million). The number of paired end reads in each RNA-Seq library, including the SHR/OlaIpcv libraries, is provided in Supplementary Table S1.

The median strain-specific genome alignment rate of the 90 HXB/BXH RI panel RNA-Seq libraries was 97% (interquartile range = 96.8–97.5%) and the alignment rate ranged from 79 to 98%. The alignment rate of each RNA-Seq library, including the 10 SHR/OlaIpcv libraries, is provided in Supplementary Table S2.

### 3.2 Brain Specific Transcriptome Generation for the HXB/BXH Recombinant Inbred Rat Panel

#### 3.2.1 Comparison of the Number of Isoforms per Gene in the Ensembl, StringTie, Aptardi, and Detected Above Background Transcriptomes

The transcriptome reconstruction pipeline that we have utilized consists of four steps. In the first three steps, novel transcripts are identified within the transcriptome. The fourth and final step eliminates transcripts which do not meet the threshold for detection above background. Of the 40,772 initial rat Ensembl transcripts, only 17,028 (42%) were included in the DABG transcriptome (Table 1). In the DABG transcriptome, the majority (40.5%) of transcripts were derived using reconstruction methods implemented in StringTie. There were slightly more aptardi transcripts in the DABG transcriptome than Ensembl transcripts. In summary, 71.5% of the transcripts in the DABG transcriptome were computationally derived and are not in the Rat Ensembl Transcriptome (v.99). By including the computational pipeline for transcriptome reconstruction, the Transcript:Gene ratio increased from 1.25 to 3.06. Furthermore, 83% of genes in the reference Ensembl transcriptome were annotated as though they produce a single transcript, i.e., genes without documented alternative splicing or alternative polyadenylation isoforms (Supplementary Figure S5A). In contrast, only 44% of genes in the DABG transcriptome were found to express a single transcript in brain of the HXB/BXH RI rat population (Supplementary Figure S5B).

**TABLE 1:** Summary of the number genes and transcripts generated/retained at each step in the transcriptome reconstruction pipeline. The percent of total transcripts identified by each source at each step is shown in parenthesis. The rat Ensembl reference transcriptome (v.99) was used as input for StringTie, along with whole brain RNA sequencing data, to characterize alternative splicing in brain of the HXB/BXH recombinant inbred rat panel. RNA sequencing data was likewise used, in conjunction with DNA sequence of the rat reference Rnor_6.0/rn6, to identify brain-specific alternative polyadenylation events in the HXB/BXH recombinant inbred rat panel. Finally, transcripts were filtered based on their expression estimates as determined by RSEM to retain only transcripts with substantial expression in the detected above background (DABG) transcriptome.

Dataset	Transcriptome generation step	Number of genes	Number of transcripts	Transcript:Gene ratio	Number of Ensembl transcripts	Number of StringTie transcripts	Number of aptardi transcripts
Ensembl	Step 1	32,586	40,772	1.25	40,772 (100.0%)	—	—
StringTie	Step 2	33,649	83,920	2.49	40,754 (48.6%)	43,166 (51.4%)	—
Aptardi	Step 3	33,649	155,677	4.63	40,754 (26.2%)	43,166 (27.7%)	71,757 (46.1%)
DABG	Step 4	19,517	59,751	3.06	17,028 (28.5%)	24,219 (40.5%)	18,504 (31.0%)

#### 3.2.2 Evaluation of 3′Termini

Since this is one of the first demonstrations of the integration of aptardi into the transcriptome reconstruction pipeline in a dataset of this magnitude, it was important to examine, in detail, the 3′ termini of the resulting transcriptome. To examine the performance of this pipeline we 1) compared the number of transcript/3′ terminus pairs in the aptardi transcriptome (when all possible pairs are enumerated) to the number of pairs that remained in DABG transcriptome, i.e., could we use read counts to determine the most appropriate transcript/3′ terminus pairs, and 2) compared aptardi-identified polyadenylation sites to annotated polyadenylation sites *via* the Ensembl transcriptome or the StringTie reconstruction.

Filtering transcript/3′ terminus pairs using our computational pipeline dramatically reduced the number of transcripts with which a 3′ terminus was associated. The aptardi analysis identified 71,757 new transcript/3′ terminus pairs (i.e., aptardi transcripts). These represent 34,003 unique 3′ termini resulting in each unique 3′ terminus being associated with approximately two transcripts on average. After filtering to yield the DABG transcriptome, there were 18,504 aptardi transcripts representing 14,388 3′ termini (approximately 1.3 aptardi transcripts associated with each 3′ terminus).

Within this pipeline, aptardi analyzes the 3′ end of all transcripts. Because of this, it is possible for aptardi to identify a 3′ terminus in a transcript/3′ terminus pair that has been identified using another source (i.e., Ensembl or StringTie). Of the initial 71,757 aptardi transcripts, 19% matched the 3′ terminus of an Ensembl or StringTie transcript +/− 100 bases, i.e., the 3′ terminus was not novel but had not been paired with that particular transcript before. When the percent of aptardi transcripts that matched the 3′ terminus of an Ensembl or StringTie transcript +/− 100 bases was calculated based on the number of aptardi 3′ termini rather than the number of aptardi transcripts (i.e., aptardi transcripts with matching 3′ termini were only counted once), a similar percentage was observed (21%; Supplementary Table S3). Of the 18,504 DABG aptardi transcripts, 16% had a 3′ terminus that matched annotation (+/− 100 bases) from the Ensembl reference or StringTie transcriptome (Supplementary Table S3). The slight decrease in the number of aptardi 3′ termini matching a StringTie or Ensembl 3′ terminus in the DABG transcriptome compared to pre-filtering suggests the filtering removes some false positive aptardi transcript/3′ terminus pairs in favor of the original StringTie/Ensembl transcript with the given polyadenylation site. At the same time, the relatively high number of overlapping Ensembl/StringTie and aptardi 3′ termini in the DABG transcriptome suggests that aptardi annotation of the polyadenylation site for the given transcript is accurate and simply represents a transcript with a similar 3′ terminus to another StringTie/Ensembl transcript with a different upstream exon structure.

#### 3.2.3 Heritability of Transcripts in the Detected Above Background Transcriptome

**FIGURE 1:**
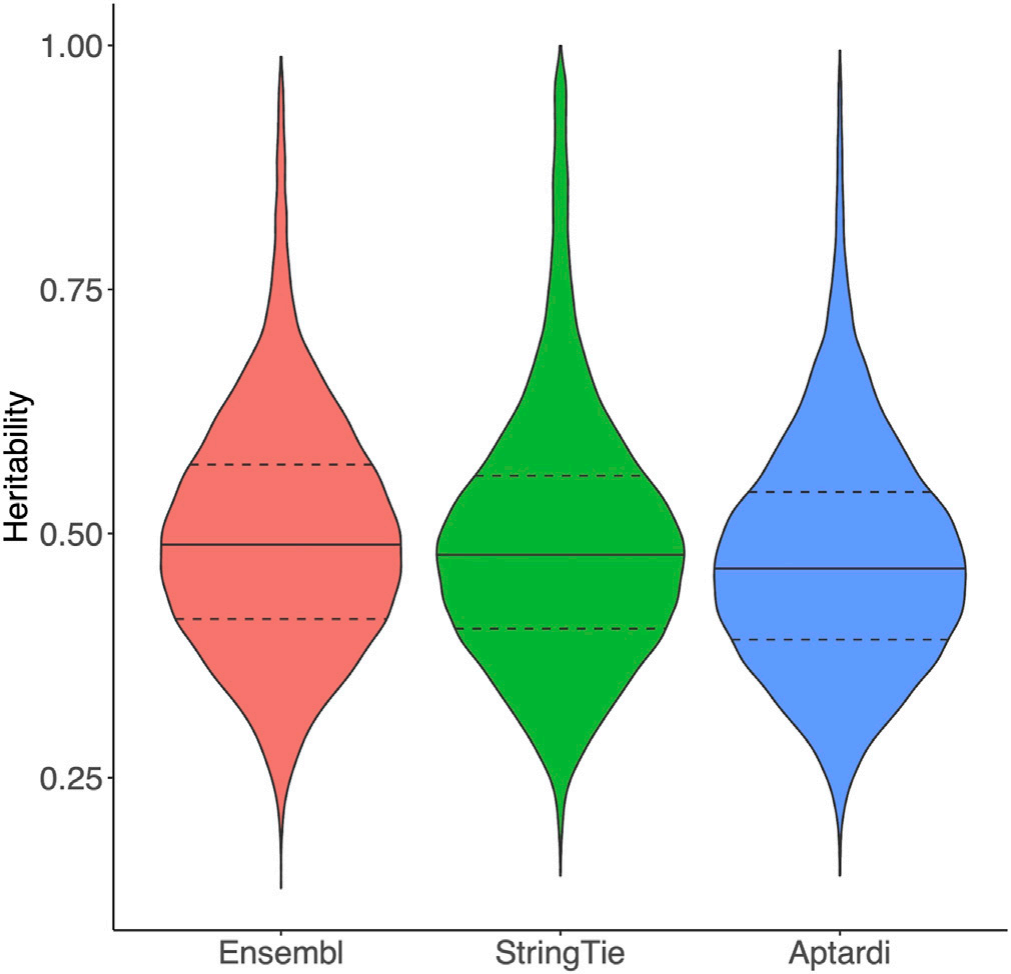
Heritability of transcripts in the detected above background transcriptome. Heritability of transcripts derived from the Ensembl reference annotation, StringTie, and aptardi. Heritability was estimated as the coefficient of determination (R^2^) from a one-way ANOVA of individual rat expression values using strain as the predictor (30 strains total) and transcript normalized expression estimates as the response. The solid horizontal line within each violin plot represents the median heritability for that group of transcripts. The dashed horizontal lines represent the upper quartile and lower quartile heritabilities for that group of transcripts.

Transcripts in the DABG transcriptome identified by Ensembl, StringTie, or aptardi displayed similar heritability to one another (Figure 1).

### 3.3 Voluntary Alcohol Consumption Quantitative Trait Loci

**FIGURE 2:**
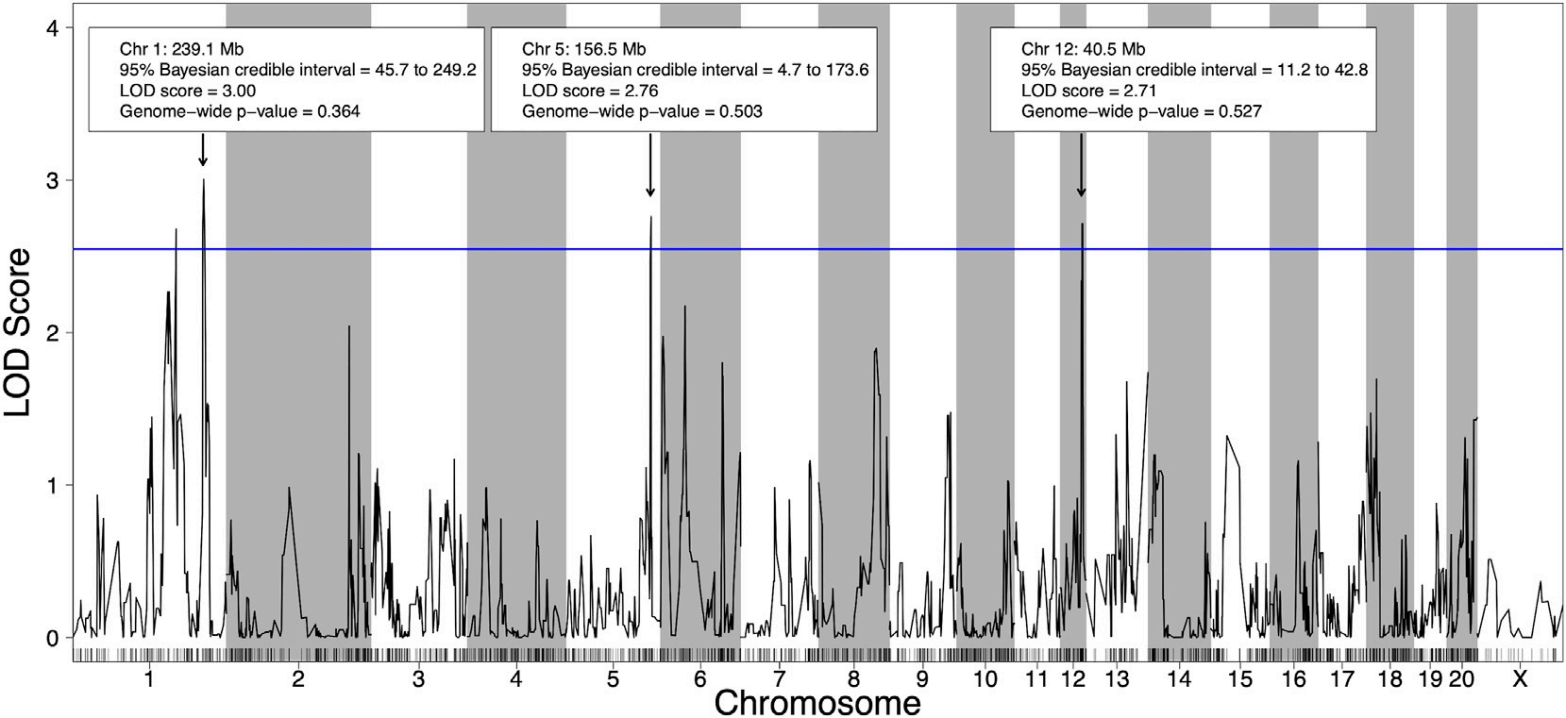
Quantitative trait loci (QTL) for voluntary alcohol consumption in the HXB/BXH recombinant inbred panel. Strain means were used in a marker regression to determine phenotypic QTL. The blue line represents the logarithm of the odds (LOD) score threshold for a suggestive QTL (genome-wide *p*-value = 0.63). Suggestive QTL are labeled with their location, 95% Bayesian credible interval, LOD score, and genome-wide *p*-value. Empirical genome-wide phenotypic QTL *p*-values were calculated using 1,000 permutations.

Using the 21 HXB/BXH RI strains with voluntary alcohol consumption data and genotype data (Supplementary Table S4), four suggestive (*p*-value < 0.63) pQTL for voluntary alcohol consumption were identified; two on chromosome 1, one on chromosome 5 and one on chromosome 12 (Figure 2). To deduce if the two suggestive peaks on chromosome 1 represented independent QTLs, a second QTL analysis was done that included the maximum peak on chromosome 1 as a covariate (Supplementary Figure S6). Since the second QTL analysis did not include any QTL on chromosome 1, the two peaks on chromosome 1 likely represent regions in linkage disequilibrium and were treated as a single QTL in the remainder of the analysis. Notably, the other pQTL on chromosomes 5 and 12 remained suggestive in the second QTL analysis, indicating these pQTL are independent of the pQTL on chromosome 1.

### 3.4 Identification of Candidate Coexpression Networks and Candidate Individual Transcripts Associated With Voluntary Alcohol Consumption

#### 3.4.1 Candidate Individual Transcripts

Of the 18,534 individual transcripts whose strain mean normalized expression estimates were subjected to correlation analysis with strain mean voluntary alcohol consumption, 64 were significantly (*p*-value < 0.01) correlated with voluntary alcohol consumption. Requiring a significant (genome-wide *p*-value < 0.01) eQTL, as well as eQTL overlap with a pQTL (using 95% Bayesian credible intervals) resulted in a final set of 11 transcripts (Table 2). One of these transcripts was identified by aptardi, six were identified by StringTie, and four were identified by Ensembl. Seven of the 11 transcripts belonged to genes expressing multiple isoforms. All 11 transcripts possessed local eQTL with the exception of the transcript from the *Map3k7* gene (*MSTRG.23809.1*).

**TABLE 2:** Individual candidate transcripts in brain for predisposition to voluntary alcohol consumption. Strain mean normalized expression estimates and strain mean voluntary alcohol consumption values were used to determine Spearman’s rank correlation coefficients. Strain mean normalized expression estimates were used in a marker regression to determine expression quantitative trait loci (eQTL) and corresponding logarithm of the odds (LOD) scores. Empirical genome-wide expression QTL *p*-values were calculated using 1,000 permutations. Transcripts are ordered by their *p*-value related to correlation with alcohol consumption. The total number of transcripts generated from the same gene in the detected above background transcriptome, and the source(s) identifying the transcripts, is also shown. All transcripts possessed local eQTL with the exception of the transcript from the *Map3k7* gene (*MSTRG.23809.1*).

Transcript ID	Gene symbol(s)	Gene description(s)	Source	Transcript correlation with alcohol consumption (correlation coefficient (*p*-value)]	Expression QTL LOD score (genome-wide (*p*-value)]	Expression QTL (chromosome: Position (Mb)]	Number of brain transcripts identified in HXB/BXH RI panel	Number of Ensembl/StringTie/aptardi transcripts
ENSRNOT00000072618	*E2f2*	E2F transcription factor	Ensembl	−0.66 (0.0013)	13.88 (<0.001)	5:154.8	1	1/0/0
MSTRG. 1868.13	*Tmem9b*	Transmembrane protein 9b	StringTie	−0.62 (0.0030)	5.26 (<0.001)	1:173.9	11	0/10/1
MSTRG.1793.4	*Trim68*	Tripartite motif-containing 68	StringTie	0.61 (0.0031)	13.06 (<0.001)	1:167.2	4	3/1/0
ENSRNOT00000075003	*Tmem159*	Transmembrane protein 159	Ensembl	−0.60 (0.0038)	9.33 (<0.001)	1:189.2	1	1/0/0
MSTRG.23809.1	*Map3k7*	Mitogen activated protein kinase kinase kinase 7	StringTie	0.60 (0.0041)	5.92 (0.004)	5:46.8	2	1/1/0
ENSRNOT00000090867	*Oas3*	2′-5′-oligoadenylate synthase 3	Ensembl	0.60 (0.0043)	9.29 (<0.001)	12:40.5	1	1/0/0
ENSRNOT00000024093.1	*Aldh1a7*	Aldehyde dehydrogenase, cytosolic 1	aptardi	−0.59 (0.0051)	14.28 (<0.001)	1:237.6	1	0/0/1
ENSRNOT00000001752	*P2rx4*	Purinergic receptor P2X 4	Ensembl	−0.58 (0.0059)	9.81 (<0.001)	12:39.1	2	1/1/0
MSTRG. 1874.1	*Tmem41b*	Transmembrane protein 41B	StringTie	0.57 (0.0068)	9.37 (<0.001)	1:173.9	3	1/2/0
MSTRG. 2084.2	*Lat, Spns1, Nfatc2ip*	Linker for activation of T-cells family member 1, Protein spinster homolog 1, NFATC2-interacting protein	StringTie	0.56 (0.0089)	7.65 (<0.001)	1:197.0	7	1/6/0
MSTRG.1526.1	*Pex11a*	Peroxisomal membrane protein 11A	StringTie	0.55 (0.0093)	11.02 (<0.001)	1:141.0	2	1/1/0

##### 3.4.1.1 Map3k7

**FIGURE 3:**
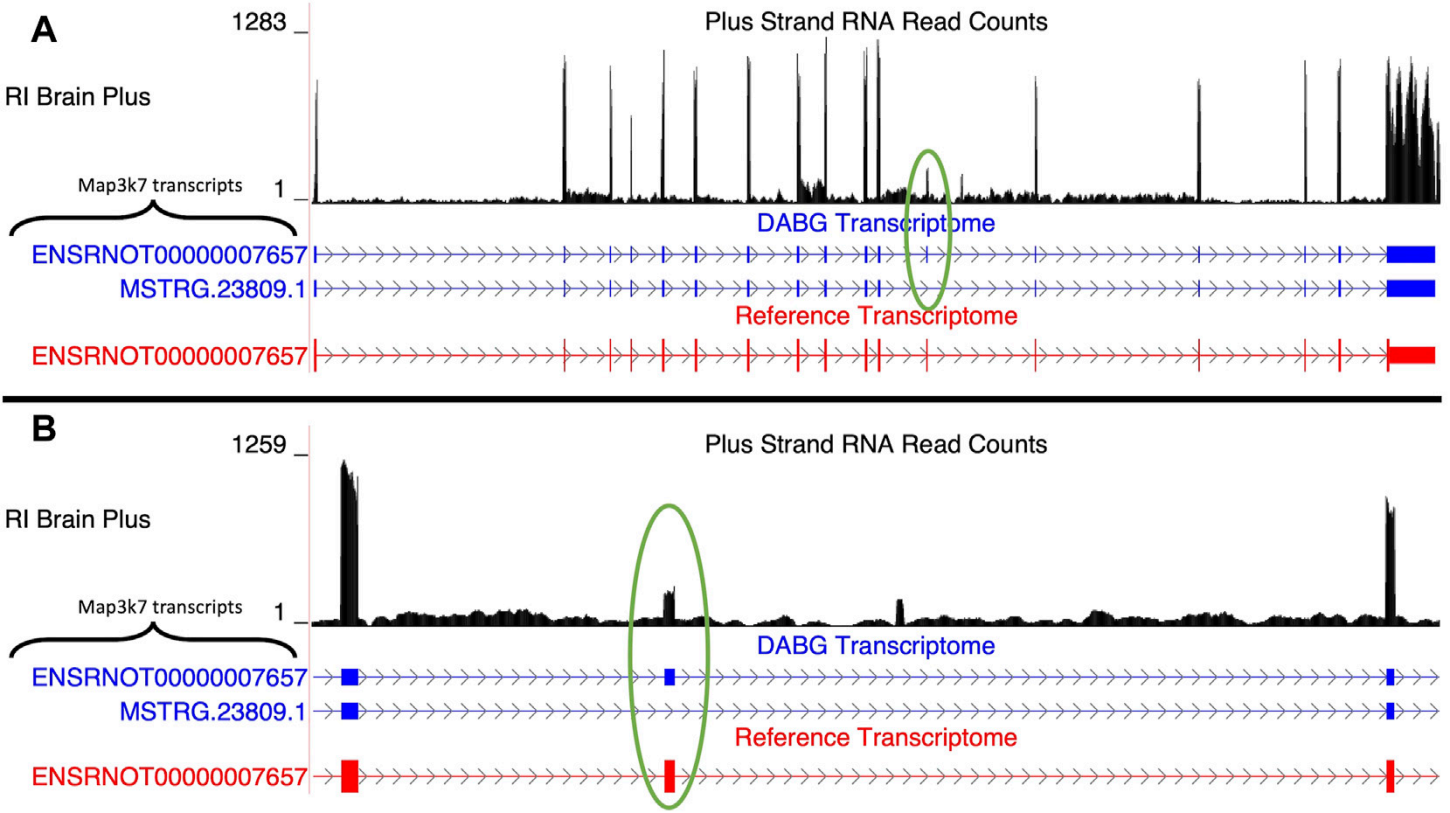
Isoforms of the *Map3k7* gene. **(A)** Blue transcripts represent those identified in the detected above background (DABG) transcriptome, and the red transcript represents the transcript present in the Ensembl reference annotation. *ENSRNOT00000007657* is annotated in the Ensembl reference transcriptome and retained in the DABG transcriptome. *MSTRG.23809.1* represents a novel isoform identified by StringTie. *MSTRG.23809.1* represents an isoform with exon 12 skipped compared to *ENSRNOT00000007657* (circled in green). **(B)** Detailed view of the exon 12 region. The RNA sequencing reads on the positive strand (black plot) represent a 10% randomly sampled subset from the HXB/BXH recombinant inbred rat panel RNA sequencing data in brain. This image was generated using the UCSC Genome Browser (Kent et al., 2002).

Two isoforms of Map3k7 (gene ID = *MSTRG.23809*) were present in the DABG transcriptome (Figure 3A), of which one was from the rat Ensembl reference transcriptome (*ENSRNOT00000007657*) and one was identified by StringTie (*MSTRG.23809.1*). *MSTRG.23809.1*, the transcript associated with alcohol consumption, is located on the plus strand of chromosome 9 (114.02–114.07 Mb), but its eQTL overlapped the voluntary alcohol consumption pQTL on chromosome 5. The transcript structure of *MSTRG.23809.1* represents an exon skipping isoform of *ENSRNOT00000007657*; specifically, it lacks exon 12 (Figure 3B). No transcripts for Map3k7 were detected in the single molecule RNA sequencing. Across the 63 individual rat RNA-Seq libraries (21 HXB/BXH RI strains) with voluntary alcohol consumption data, the mean transcripts per million transcripts (TPM) of *MSTRG.23809.1* and *ENSRNOT00000007657* was 0.95 and 1.30, respectively.

We note that a transcript with a nearly identical transcript sequence as *MSTRG.23809.1* was identified *de novo* by StringTie (*MSTRG.17584.2*) on the plus strand of chromosome 5 (47.19–47.24 Mb), which overlaps the voluntary alcohol consumption pQTL. The two transcripts only differed in the lengths of their untranslated regions (less than 10 nucleotides in the 5′ UTR and approximately 200 nucleotides in the 3′ UTR). In fact, the two transcripts have identical nucleotide sequences in the largest open reading frame of each transcript. When the transcript sequences were aligned to the genome, both transcripts align perfectly to both genomic regions. While the gene of *MSTRG.17584.2* (*LOC100910771*) is annotated as *Map3k7-like* in both Ensembl v.99 and the latest release (v.104), the Rat Genome Database has since retired the *Map3k7-like* annotation in favor of *Map3k7*. Therefore, there are multiple locations of this gene/transcript currently annotated in rat according to the Rat Genome Database. The two transcripts are highly negatively correlated (Spearman’s rank correlation coefficient = −0.835), and both transcripts’ strain mean normalized expression estimates have a similar (absolute) Spearman’s rank correlation coefficient to strain mean voluntary alcohol consumption (*MSTRG.23809.1* = 0.599; *MSTRG.17584.2* = -0.509), but the slightly weaker correlation of *MSTRG.17584.2* caused it to be removed as a candidate transcript based on correlation *p*-value (*p*-value = 0.018). Yet its eQTL (chromosome 5, position = 46.8 Mb, 95% Bayesian credible = 46.8–46.8 Mb) overlapped the alcohol pQTL on chromosome 5, thus meeting this criterion for candidacy and, with respect to the eQTL peak, the LOD score and corresponding genome-wide *p*-value were more significant for *MSTRG.17584.2* (LOD = 8.72, *p*-value < 0.001) than *MSTRG.23809.1* (LOD = 5.92, *p*-value = 0.004). Also of note, the eQTL for *MSTRG.17584.2* overlaps its genomic position (i.e., is a local eQTL), whereas *MSTRG.17584.2* possesses a distal eQTL. According to our quantitation, *MSTRG.17584.2* is the dominant transcript (mean TPM = 2.84 vs 0.95).

##### 3.4.1.2 Aldh1a7

**FIGURE 4:**
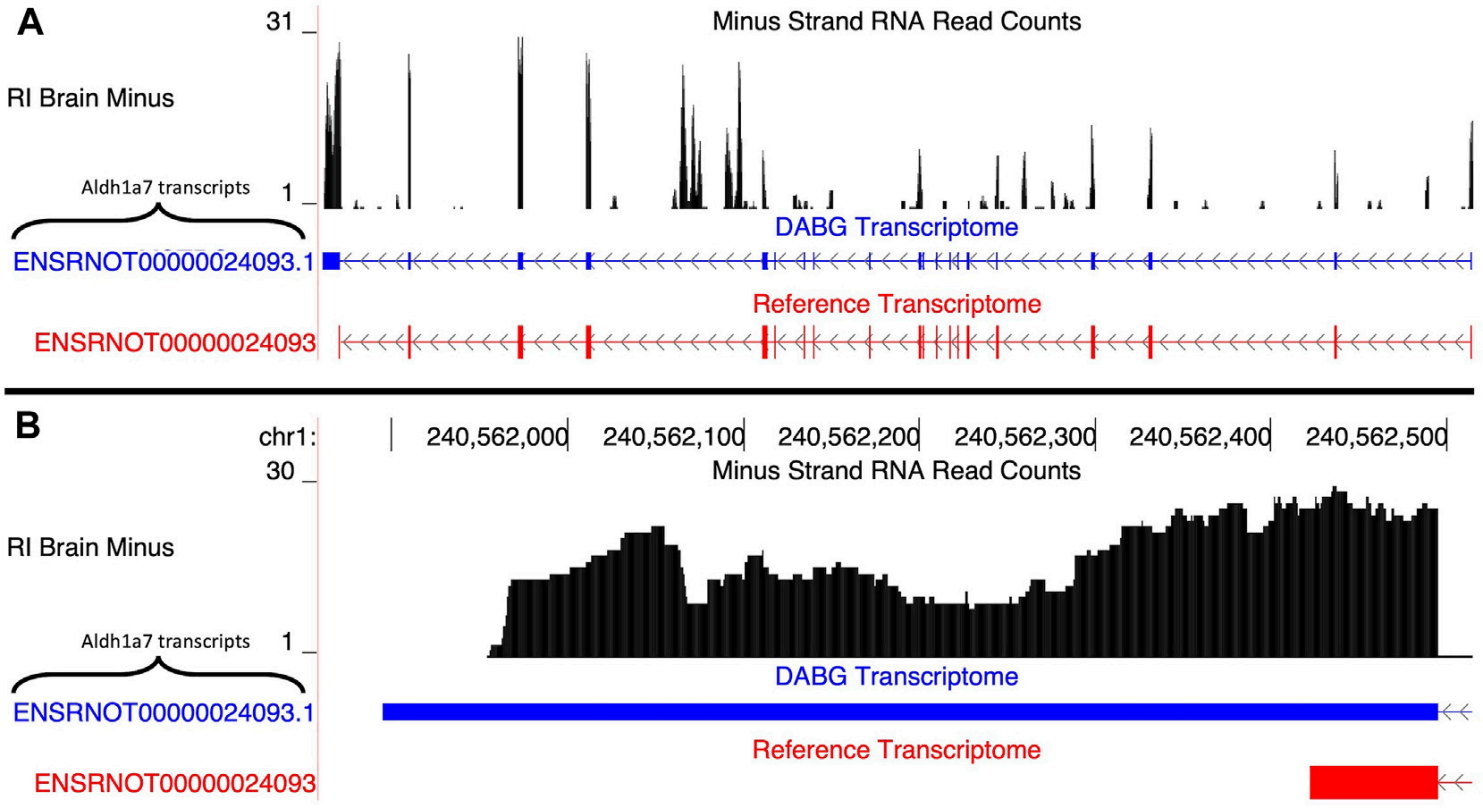
Isoforms of the *Aldh1a7* gene. **(A)** The blue transcript represents those identified in the detected above background (DABG) transcriptome, and the red transcript represents the transcript present in Ensembl reference annotation. *ENSRNOT00000024093* is annotated in the Ensembl reference transcriptome but was filtered out of the DABG transcriptome. *ENSRNOT00000024093.1* represents a novel isoform identified by aptardi. **(B)** Detailed view of the 3′ region comparing aptardi annotation (*ENSRNOT00000024093.1*) to Ensembl reference annotation (*ENSRNOT00000024093*). *ENSRNOT00000024093* and *ENSRNOT00000024093.1* differ only in the length of their 3′ most exon. The RNA sequencing reads on the negative strand (black plot) represent a 10% randomly sampled subset from the HXB/BXH recombinant inbred rat panel RNA sequencing data in brain. This image was generated using the UCSC Genome Browser (Kent, 2002).

A single transcript of *Aldh1a7*, *ENSRNOT00000024093.1*, was present in the DABG transcriptome (Figure 4A). The Ensembl version of this transcript, *ENSRNOT00000024093*, was removed during filtering and thus not present in the DABG transcriptome. The Ensembl transcript was removed after the first quantitation step that generated the high-quality transcriptome from the aptardi transcriptome (i.e., it was removed because it had estimated read counts of zero in one third or more of the 90 HXB/BXH RI panel libraries). *ENSRNOT00000024093.1* shares exon junctions with *ENSRNOT00000024093* (Figure 4A) but possesses a unique 3′ terminus on the negative strand of chromosome 1 compared to Ensembl (*ENSRNOT00000024093.1* 3′ end = 240,561,896; *ENSRNOT00000024093* 3′ end = 240,562,423; Figure 4B). No Aldh1a7 transcripts were detected in the single molecule RNA sequencing.

#### 3.4.2 Candidate Modules From Weighted Gene Coexpression Network Analysis

A total of 30 HXB/BXH RI strains with expression data (Supplementary Table S4) were used to generate transcript coexpression modules using strain means of transcript normalized expression estimates. WGCNA identified 215 modules along with 137 transcripts (out of the 18,543) that were not assigned a module. The median module size was 10 transcripts (Supplementary Figure S7). Module eigengenes captured much of the within-module transcript expression variability (interquartile range: 60–72%). In addition, when a gene had multiple isoforms included in the WGCNA, most often (54% of genes) isoforms generated from the same gene were assigned to different modules (Supplementary Figure S8).

Of the 215 modules, the module eigengene of a single module—blue1—was the only module significantly associated with voluntary alcohol consumption (correlation coefficient = −0.62, *p*-value = 0.0026). A significant meQTL for the blue1 module was identified on chromosome 12 (LOD score = 16.83, genome-wide *p*-value < 0.0001). Furthermore, the location of the meQTL (chromosome 12, position = 39.1 Mb, 95% Bayesian credible = 39.1–40.5 Mb) overlapped the suggestive pQTL on chromosome 12 (chromosome 12, position = 40.5 Mb, 95% Bayesian credible = 11.2–42.8 Mb) thereby satisfying all the requirements for candidacy. The module eigengene explained 75% of the within-module expression variability.

**FIGURE 5:**
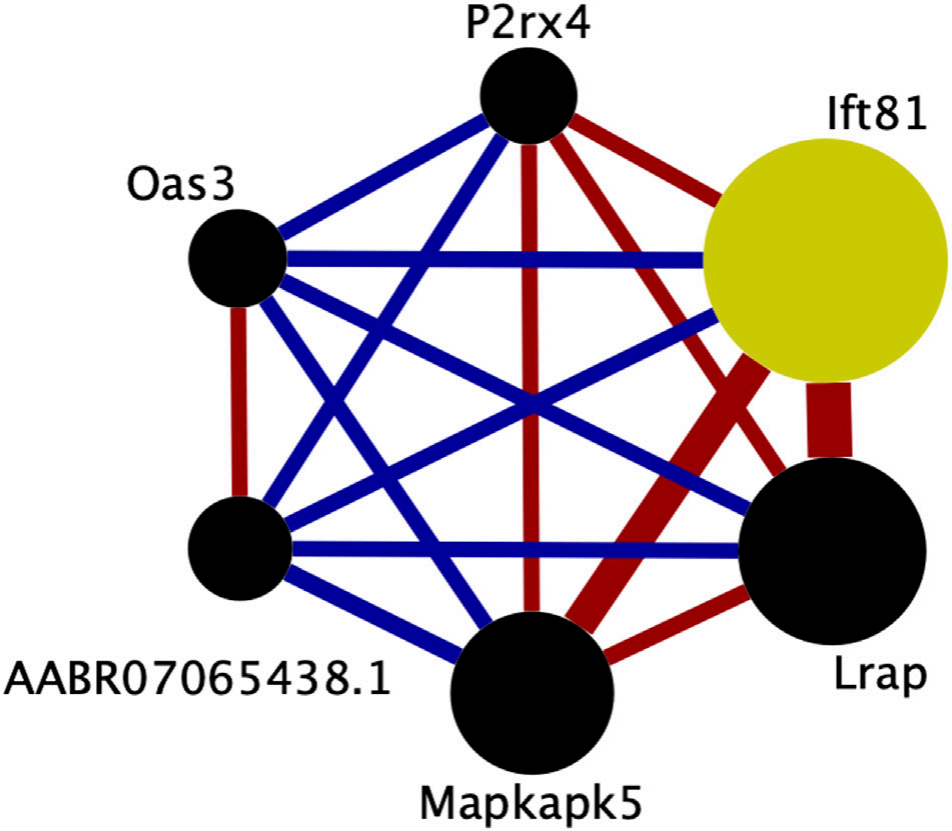
Connectivity within the brain candidate coexpression module for predisposition to voluntary alcohol consumption. Each circle represents a transcript from the coexpression module. The size of each circle is weighted based on its intra-modular connectivity (not to scale), and the thickness of each edge is weighted based on the magnitude of the connectivity between the two transcripts (not to scale). The edge colors indicate the direction of the connectivity (red = positive, blue = negative). Transcripts are ordered by intra-modular connectivity clockwise starting with Ift81. Strain mean normalized transcript expression estimates were used in weighted gene coexpression network analysis to generate coexpression modules. This figure was generated using Cytoscape (v. 3.8.2) (Shannon et al., 2003).

The transcripts comprising the blue1 module are shown in Figure 5 and listed in Table 3. One transcript was identified by aptardi (*Ift81*), three were identified by StringTie (*Lrap*, *Mapkapk5*, *AABR07065438.1*) and two were in the original Ensembl annotation (*P2rx4* and *Oas3*). Most of these transcripts reside near the physical location of the meQTL and pQTL. The expression of all individual transcripts in the blue1 module displayed correlation with voluntary alcohol consumption (*p*-value < 0.05). Three of the six transcripts were associated with genes that had more than one transcript (*Mapkapk5*, *P2rx4*, and *Ift81*) in the DABG transcriptome, although not all of these transcripts were included in WGCNA after applying the computational pipeline that removes lowly expressed transcripts. The genes of three transcripts (*Lrap*, *P2rx4* and *Ift81*) were also identified in our previous candidate module (Saba et al., 2015) using microarray data (vs. RNA-Seq data here).

**TABLE 3:** Transcripts in the brain candidate coexpression module for predisposition to voluntary alcohol consumption. Transcripts are ordered by intra-modular connectivity, and the source that identified the transcript is shown. The total number of transcripts with the same gene ID as the individual candidate transcript (i.e., isoforms of the same gene) in the detected above background transcriptome, and the source(s) identifying the transcripts, is also shown. Strain mean normalized expression estimates and strain mean voluntary alcohol consumption values were used to determine Spearman’s rank correlation coefficients.

Transcript ID	Gene symbol	Gene description	Source	Chromosome: Start position-end position (Mb) (strand)	Intra-modular connectivity	Transcript correlation with alcohol consumption (correlation coefficient (*p*-value)]	Number of transcripts identified in HXB/BXH RI panel	Number of ensembl/StringTie/aptardi transcripts
ENSRNOT00000066952.1	Ift81	Intraflagellar transport 81	aptardi	12:39.42–39.51 (+)	1.22	−0.44 (0.0487)	3	0/1/2
MSTRG.6250.1	Lrap	Locus regulating alcohol preference	StringTie	12:39.01–39.02 (−)	1.14	−0.45 (0.0417)	1	0/1/0
MSTRG.6281.1	Mapkapk5	MAPK activated protein kinase 5	StringTie	12:40.51–40.53 (+)	1.10	−0.49 (0.0238)	3	2/1/0
MSTRG.19929.1	AABR07065438.1	Ribosomal protein L6, pseudo 1	StringTie	6:128.74–128.74 (+)	1.01	0.51 (0.0191)	1	0/1/0
ENSRNOT00000090867	Oas3	2′-5′-oligoadenylate synthetase 3	Ensembl	12:41.32–41.34 (+)	1.01	0.60 (0.0043)	1	1/0/0
ENSRNOT00000001752	P2rx4	Purinergic receptor P2X 4	Ensembl	12:39.31–39.33 (−)	1.00	−0.58 (0.0059)	2	1/1/0

##### 3.4.2.1 P2rx4

**FIGURE 6:**
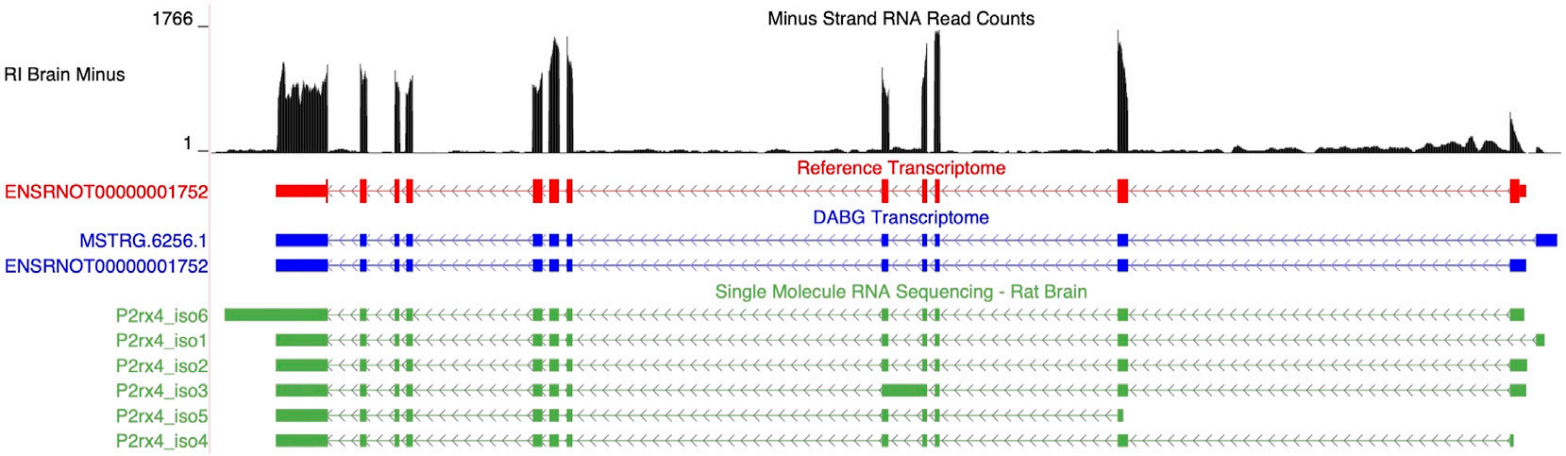
Isoforms of the *P2rx4* gene. Blue transcripts represent those identified in the detected above background (DABG) transcriptome, the red transcript represents the transcript present in Ensembl reference annotation, and the green transcripts represent transcripts identified in the single molecule RNA sequencing data from rat brain. *ENSRNOT00000001752* is annotated in the Ensembl reference transcriptome and retained in the DABG transcriptome, whereas *MSTRG.6256.1* represents a novel isoform identified here by StringTie. *MSTRG.6256.1* and *ENSRNOT00000001752* differ in their 5′ most exon but otherwise share exon junctions. The RNA sequencing reads on the negative strand (black plot) represent a 10% randomly sampled subset from the HXB/BXH recombinant inbred rat panel RNA sequencing data in brain. This image was generated using the UCSC Genome Browser (Kent et al., 2002).

Two isoforms of *P2rx4* (gene ID = *MSTRG.6256*) were identified in the DABG transcriptome: *ENSRNOT00000001752*, which was annotated in the Ensembl transcriptome and included in the candidate module, and *MSTRG.6256.1*, which was annotated by StringTie (Figure 6). The Ensembl and StringTie transcripts differ at their 5′ exon but otherwise share identical transcript structure. Both were included in WGCNA but belonged to different coexpression networks. Six isoforms of *P2rx4* were identified in the single molecule RNA sequencing. The isoform labeled P2rx4_iso2 in Figure 6 matches *ENSRNOT00000001752* while P2rx4_iso1 validates the novel splice variant identified by StringTie.

*ENSRNOT00000001752* was also identified as a candidate transcript (Table 2). While the strain mean normalized expression estimates of *ENSRNOT00000001752* were negatively correlated with strain mean voluntary alcohol consumption (Spearman’s rank correlation = −0.579, *p*-value = 0.0051), *MSTRG.6256.1* was not significantly associated with alcohol consumption (Spearman’s rank correlation = 0.166, *p*-value = 0.471). *ENSRNOT00000001752* was the dominant isoform in the 63 individual rat RNA-Seq libraries (21 HXB/BXH RI strains) with voluntary alcohol consumption data (*ENSRNOT00000001752* mean TPM = 6.143; *MSTRG.6256.1* mean TPM = 0.254).

##### 3.4.2.2 Ift81

**FIGURE 7:**
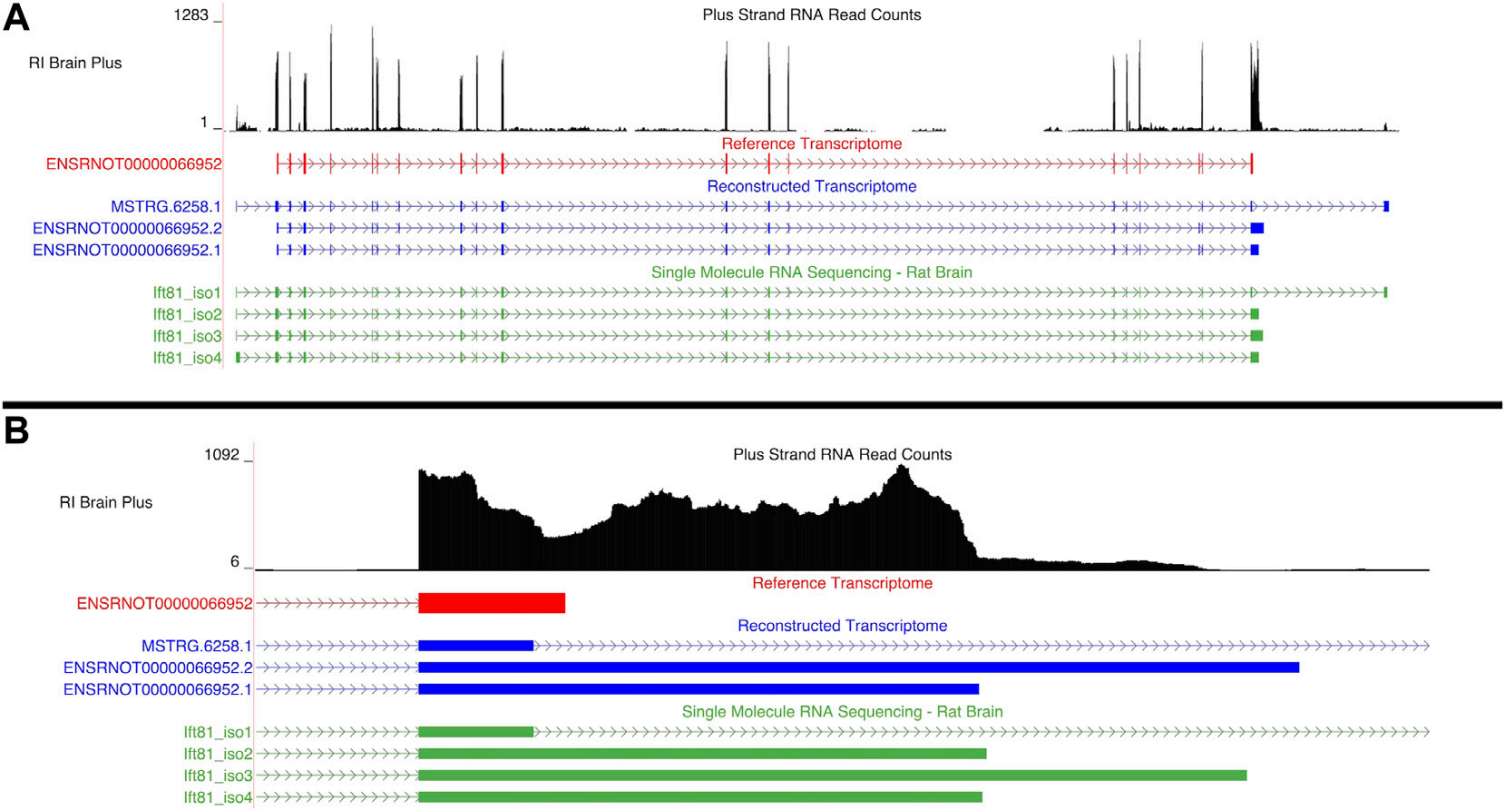
Isoforms of the *Ift81* gene. **(A)** Blue transcripts represent those identified in the detected above background (DABG) transcriptome, the red transcript represents the transcript present in Ensembl reference annotation, and the green transcripts represent transcripts identified in the single molecule RNA sequencing data from rat brain. *ENSRNOT00000066952* is annotated in the Ensembl reference transcriptome but removed in the DABG transcriptome. *MSTRG.6258.1* represents a novel isoform identified by StringTie, and *ENSRNOT00000066952.1* and *ENSRNOT00000066952.2* represent novel isoforms identified by aptardi. **(B)** Detailed view of the 3′ region comparing aptardi annotation (*ENSRNOT00000066952.1* and *ENSRNOT00000066952.2*) to Ensembl annotation (*ENSRNOT00000066952*). *ENSRNOT00000066952*, *ENSRNOT00000066952.1*, and *ENSRNOT00000066952.2* differ only in the length of their 3′ most exon, whereas *MSTRG.6258.1* possesses a different exon structure. The RNA sequencing reads on the positive strand (black plot) represent a 10% randomly sampled subset from the HXB/BXH recombinant inbred rat panel RNA sequencing data in brain. This image was generated using the UCSC Genome Browser (Kent et al., 2002).

An aptardi isoform of *Ift81*, *ENSRNOT00000066952.1*, was the hub transcript, (i.e., the transcript with the highest intra-modular connectivity within the blue1 module). Two additional isoforms of this gene were annotated in the DABG transcriptome: *ENSRNOT00000066952.2*, which was annotated by aptardi, and *MSTRG.6258.1*, which was identified by StringTie (Figure 7A). The Ensembl annotation of *Ift81*, *ENSRNOT00000066952*, was not present in the DABG transcriptome. Like *Aldh1a7*, the Ensembl transcript for *Ift81* was removed after the first quantitation step that generated the high-quality transcriptome (i.e., it was removed because it had zero estimated read counts in one third or more of the 90 HXB/BXH RI panel libraries). Both aptardi transcripts only differ in their 3′ base position (on the plus strand of chromosome 12) compared to the Ensembl transcript (*ENSRNOT00000066952* 3′ end = 39,506,890; *ENSRNOT00000066952.1* 3′ end = 39,507,407; *ENSRNOT00000066952.2* 3′ end = 39,507,807; Figure 7B). In contrast, the transcript identified by StringTie, *MSTRG.6258.1*, possesses unique exon structure (Figure 7A). The unique exon structure of *MSTRG.6258.1* was validated using the single molecule RNA sequencing (Ift81_iso1 in Figure 7A). The alternative 3′ ends were also validated in the single molecule RNA sequencing. The 3′ ends of Ift81_iso2 and Ift81_iso4 were less than 10 basepairs from the 3′ end of *ENSRNOT00000066952.1* and the 3′ end of Ift81_iso3 66 basepairs shorter than the 3′ end of *ENSRNOT00000066952.2*.

The isoform assigned to the blue1 module (*ENSRNOT00000066952.1*) was the only individual isoform of this gene significantly associated with voluntary alcohol consumption (*ENSRNOT00000066952.1*: correlation = −0.44, *p*-value = 0.049; *ENSRNOT00000066952.2*: correlation = −0.33, *p*-value = 0.14, *MSTRG.6258.1*: correlation = −0.15, *p*-value = 0.52). Across the 63 RNA-Seq samples of the 21 HXB/BXH RI strains with voluntary alcohol consumption data, the mean TPM was 0.68, 2.35, and 2.67 for *ENSRNOT00000066952.2*, *ENSRNOT00000066952.1*, and *MSTRG.6258.1*, respectively. Only *ENSRNOT00000066952.1*, and *MSTRG.6258.1* were subjected to WGCNA due to the computational pipeline that filtered lowly expressed transcripts prior to WGCNA.

##### 3.4.2.3 Mapkapk5

The DABG transcriptome contained three isoforms for the *Mapkapk5* gene (gene ID = *MSTRG.6281*), two of which were annotated by Ensembl (*ENSRNOT00000065314*, *ENSRNOT00000001817*) and one that was identified by StringTie (*MSTRG.6281.1*). The StringTie transcript represents the transcript in the candidate module; both Ensembl transcripts were removed during the DABG transcriptome generation process due to heritability values below the median, and thus these transcripts were not subjected to WGCNA. *MSTRG.6281.1* shares similar exons to the longer Ensembl isoform *ENSRNOT00000001817* but with a noticeably longer 3′ terminal exon and an additional, long 5′ exon (Supplementary Figure S9). The alternative 3′ terminal exon was not observed in the single molecule RNA sequencing data. The mean TPM values across the 63 RNA-Seq samples of the 21 HXB/BXH RI strains with voluntary alcohol consumption data were 1.29, 1.46, and 8.25 for *ENSRNOT00000065314*, *MSTRG.6281.1*, and *ENSRNOT00000001817*, respectively. The strain mean normalized expression estimates of *MSTRG.6281.1* displayed the greatest individual association with voluntary alcohol consumption (correlation = −0.49, *p*-value = 0.024) compared to the Ensembl transcripts (*ENSRNOT00000065314*: correlation = 0.17, *p*-value = 0.46, *ENSRNOT00000001817*: correlation = 0.39, *p*-value = 0.081).

## 4 Discussion

### 4.1 Characterization of the Brain Specific Transcriptome in the HXB/BXH Recombinant Inbred Rat Panel

A major goal of this work was to annotate the brain specific transcriptome in the HXB/BXH recombinant inbred rat panel by applying computational methods—namely StringTie and aptardi—that incorporate expression data (in the form of RNA-Seq). By including expression data, we were able to characterize the expressed transcriptome under our specific study conditions. Moreover, our deeply sequenced RNA-Seq libraries provided ample data for transcript identification and quantitation. For this manuscript, we chose to focus our RNA analyses to only strains from the HXB/BXH recombinant inbred panel rather than the full HRDP because splice variants and alternative polyadenylation sites that are unique to either progenitor strains will likely be represented by approximately half of the RI strains. This decreases the likelihood of identifying a transcript variant that is exclusive to a single RI strain in the analysis thus increasing our power for detection. A secondary goal of this manuscript was to re-examine the relationship of alcohol consumption in the two-bottle choice paradigm to brain RNA expression using an updated and expanded transcriptome data set.

Because the rat Ensembl transcriptome is under annotated compared to humans and other species such as mouse (Ji et al., 2020), many of transcripts in the DABG transcriptome were identified by the computational approaches that utilized RNA-Seq data (Table 1) and were not included in the rat Ensembl transcriptome (v.99), highlighting the importance of annotating transcriptomes in the context of the conditions. Additionally, the comparable expression heritabilities of StringTie and aptardi transcripts to Ensembl transcripts indicates these algorithms annotate transcripts that demonstrate significant genetic influences and robust expression similar to those transcripts that are well annotated.

Unsurprisingly, we found that the transcript:gene ratio in the DABG transcriptome was much greater than existing rat Ensembl reference annotation (Table 1), and many more genes in the DABG transcriptome expressed more than one isoform compared to the Ensembl reference annotation (Supplementary Figure S5). This aligns with the literature that many genes from higher order eukaryotic organisms express alternative splicing and/or alternative polyadenylation transcripts (Nilsen and Graveley, 2010; Tian and Manley, 2017) and, in particular, that brain expresses the greatest mRNA diversity compared to other tissues due to alternative splicing and alternative polyadenylation (MacDonald, 2019). Furthermore, a recent study also observed an increase in the transcript:gene ratio in rat when including RNA-Seq data to generate the transcriptome compared to Ensembl annotation (Ji et al., 2020). In the DABG transcriptome, a significant portion of transcripts were derived from each of the three sources (i.e., StringTie, aptardi, and Ensembl), demonstrating the importance of both StringTie and aptardi in our transcript reconstruction pipeline.

Also of note, we suggest that our transcriptome generation pipeline, including the filtering procedure, provides a means to generate a high-quality, representative transcriptome. In the DABG transcriptome, many genes did not include a Ensembl transcript. We hypothesize this is because many isoforms identified by StringTie and/or aptardi more accurately annotated the transcripts expressed by those genes. Specific examples of this include *Ift81* and *Aldh1a7*, where the Ensembl transcript was removed after filtering in favor of the StringTie and/or aptardi transcripts, and these computationally identified transcripts are better supported by the RNA-Seq data (Figure 7 and Figure 4).

### 4.2 Evaluation of Genes Previously Identified as Associated With Voluntary Alcohol Consumption in the HXB/BXH Recombinant Inbred Rat Panel

We previously performed WGCNA on brain expression data in the HXB/BXH RI panel to identify networks of genes associated with the same phenotype (voluntary alcohol consumption) (Saba et al., 2015). The major difference was in the technology used to generate expression data and, as a result, the capacity to quantitate individual transcripts in the HXB/BXH RI panel. Specifically, we currently utilized RNA-Seq data (as opposed to Affymetrix exon microarray data used earlier) for the quantitative measurement of transcript expression in the full HXB/BXH RI panel. For most genes, the array was not capable of unambiguously estimating the expression of individual transcript isoforms. It is well-documented that RNA-Seq has a broader dynamic range than microarrays (Marioni et al., 2008). With RNA-Seq data from the full RI panel, we were not only able to estimate expression for each individual transcript of a gene for each individual strain, but we were also able to filter individual transcripts for their heritability across the RI panel.

The previous candidate coexpression module’s meQTL also overlapped a voluntary alcohol consumption pQTL on chromosome 12. Similarly, here we identified a single candidate coexpression module (out of 215 modules) with its meQTL/pQTL overlap also on chromosome 12. Of the six transcripts in the new candidate transcript module, genes of three were present in our previous candidate gene module—*P2rx4*, *Lrap*, and *Ift81*. Additionally, the genes of these transcripts had some of the greatest intra-modular connectivity values in the previous gene module; *Lrap*, *Ift81*, and *P2rx4* had the first, second, and fifth greatest intra-modular connectivity values, respectively.

Many other genes from the original candidate coexpression module—especially those with high intra-modular connectivity values—displayed correlation with voluntary alcohol consumption at the individual transcript level (Table 4). More specifically, we examined the genes from the previous candidate coexpression module that were either significantly associated with alcohol consumption (*p* < 0.05) in the microarray analysis or were within the top eight most highly connected genes within the original module. The RNA-Seq data from the HXB/BXH RI panel replicated the association with voluntary alcohol consumption (correlation in the same direction and *p*-value < 0.05) for four of the eight genes (Table 4). For *Txnip*, the association with alcohol was only suggestive (*p* = 0.068) in the current analysis. The two remaining genes (*Cfap91* and *Coq5*) both had two transcripts each in the RNA-Seq data and neither transcript was associated with alcohol consumption in this analysis.

**TABLE 4:** Comparison of results from microarrays to results from RNA-Seq for genes whose brain expression was previously identified as associated with voluntary alcohol consumption in the HXB/BXH recombinant inbred rat panel. Correlations were determined using Spearman’s rank correlation and strain mean gene level normalized expression estimates or strain mean transcript level normalized expression estimates and strain mean alcohol consumption values for the gene and transcript correlations, respectively. Genes are ordered by intramodular connectivity in the original microarray-based coexpression module. The number of transcripts identified for each gene in the detected above background transcriptome is shown with its source of identification. The intramodular connectivity values of genes in the candidate module from the previous study (Saba et al., 2015; Saba et al., 2020) are shown, along with their rank within the module.

Gene symbol	Gene description	From microarray data (Saba et al. (2015), Saba et al. (2020)		From HXB/BXH RNA-Seq data		
		Correlation with alcohol consumption in microarray data [correlation coefficient (*p*-Value)]	Connectivity-based intramodular connectivity (rank within module)	Number of transcripts identified in HXB/BXH panel in the DABG transcriptome	Number of Ensembl/StringTie/aptardi transcripts	Most significant transcript correlation with alcohol consumption [correlation coefficient (*p*-value)]
Lrap	Locus regulating alcohol preference	−0.55 (0.011)	2.99 (1)	1	0/1/0	−0.45 (0.042)
Ift81	Intraflagellar transport 81	−0.43 (0.051)	2.66 (2)	3	0/1/2	−0.44 (0.049)
Coq5	Coenzyme Q5, methyltransferase	−0.50 (0.021)	2.24 (3)	2	1/0/1	0.13 (0.59)
Txnip	Thioredoxin interacting protein	0.61 (0.003)	2.20 (4)	1	1/0/0	0.41 (0.068)
P2rx4	Purinergic receptor P2X 4	−0.63 (0.002)	2.15 (5)	2	1/1/0	−0.58 (0.006)
Tmem116	Transmembrane protein 116	0.34 (0.133)	2.00 (6)	1	1/0/0	0.52 (0.017)
Cfap91 (formerly Maats1)	Cilia and flagella associated protein 91	−0.56 (0.008)	1.95 (7)	2	1/0/1	0.21 (0.37)
GENE_27603	Unannotated gene	−0.51 (0.021)	1.74 (8)	Not included in DABG transcriptome		

**FIGURE 8:**
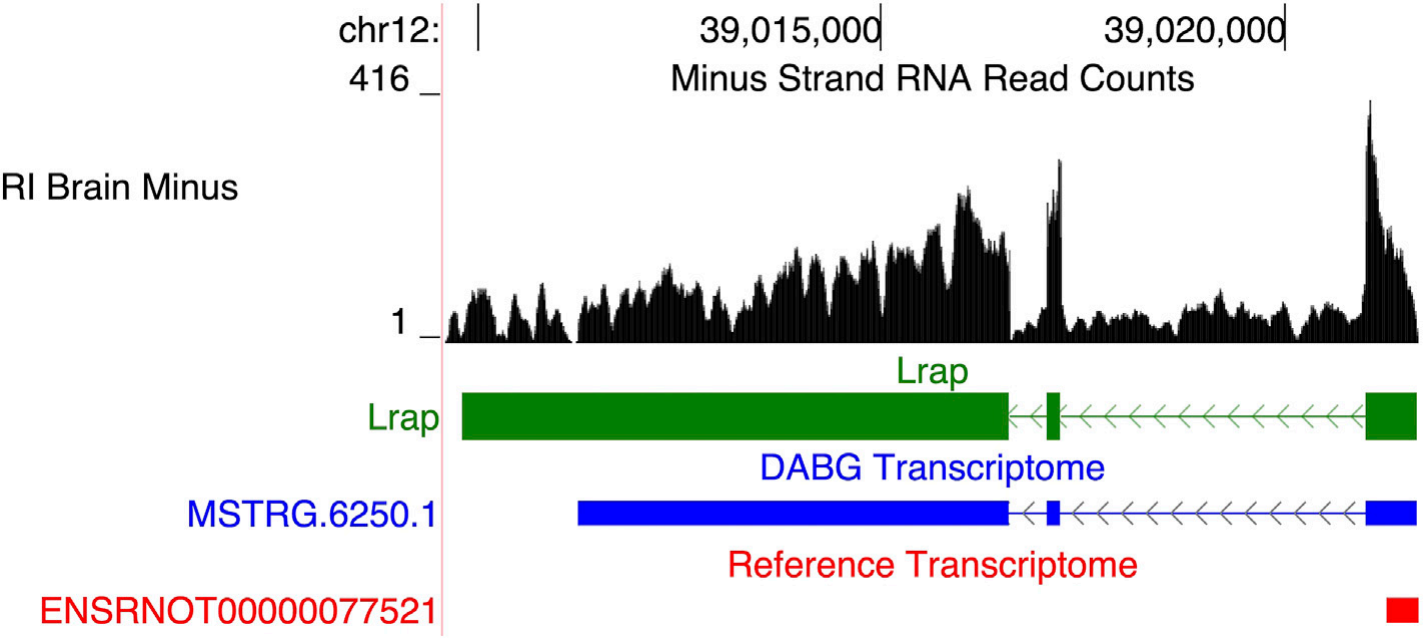
Recapitulation of *Lrap*. We previously identified a novel transcript, subsequently annotated *Lrap*, as a potential mediator of alcohol consumption that is expressed in rat brain (Saba et al., 2015; Saba et al., 2020). The structure identified previously is shown in green (*Lrap*), the *de novo* transcript identified in the detected above background (DABG) transcriptome is shown in blue (*MSTRG.6250.1*), and existing Ensembl annotation is shown in red (*ENSRNOT00000077521*). The transcript identified here as *MSTRG.6250.1* (annotated by StringTie) closely matches our previous annotation of *Lrap*. Additionally, the RNA sequencing reads on the negative strand (black plot) support the present of this transcript in our dataset. The RNA sequencing reads represent a 10% randomly sampled subset from the HXB/BXH recombinant inbred rat panel RNA sequencing data in brain. This image was generated using the UCSC Genome Browser (Kent et al., 2002).

Among the genes in the original module, a long, potentially non-coding RNA transcript, *Lrap*, was identified as the hub gene and key modulator of voluntary alcohol consumption in the HXB/BXH RI panel, initially using a systems genetics approach and subsequently using genetically manipulated rats (Saba et al., 2015, 2020). This originally unannotated transcript was initially identified using short read RNA sequencing from the progenitor strains only and its splicing structure was confirmed using PCR. In this study, we assessed whether it was present within the full HXB/BXH RI panel through our transcriptome reconstruction methods (i.e., StringTie and aptardi). A similar transcript (transcript ID = *MSTRG.6520.1*; associated gene name = AABR07036336.2)—identified by StringTie—was observed (Figure 8). *MSTRG.6250.1* was the only isoform of this gene, i.e., Gene ID *MSTRG.6520*. Both *MSTRG.6250.1* and the original *Lrap* are on the negative strand of chromosome 12 and possess three exons with similar locations in the genome (Original *Lrap*: 39,009,809–39,016,585, 39,017,055–39,017,223, and 39,021,009–39,021,641; *MSTRG.6520.1*: 39,011,243–39,016,585, 39,017,056–39,017,223, and 39,021,010–39,021,635). There are dramatic differences in the transcription stop site as expected based on the lack of precision in the initial reconstruction, yet the transcription start sites were within six base pairs of each other. The two exon junctions in *Lrap* also differed by one base pair each. Although the exon junctions were previously validated via PCR, we note that the precise locations of the 3′ and 5′ends have not been validated (Saba et al., 2015). As a result, this gene/transcript was hereafter labeled *Lrap*. With the new RNA-Seq data from the entire HXB/BXH RI panel, *Lrap* remained significantly negatively associated with alcohol consumption (correlation coefficient = −0.45; *p*-value = 0.042; Table 4). Furthermore, it was identified in the candidate coexpression network where it had the second highest intra-modular connectivity.

Overall, these results indicate, in this instance, that the major genes/transcripts in candidate coexpression modules (i.e., those with the greatest intra-modular connectivity values) are robust across diverse data acquisition methods and data analyses.

### 4.3 Candidate Coexpression Module and Candidate Individual Transcripts

#### 4.3.1 Characterization of Transcripts in the Current Candidate Coexpression Module

Existing Ensembl annotation possessed a single transcript structure for *Ift81*; here we identified three alternative transcripts (Figure 7). Moreover, filtering to yield the DABG transcriptome removed the Ensembl transcript (due to low estimated read counts), but the three new structures remained. The RNA-Seq data support the presence of these structures, exemplifying how the transcriptome generation procedure can not only annotate new transcripts, but also potentially improve annotation of existing transcripts. Furthermore, while *Ift81* was identified in the previous candidate gene module and was present in the current candidate transcript module, only a single isoform of *Ift81* was present in the current candidate transcript module, thereby enabling greater granularity as to the exact transcript that is associated with alcohol consumption. Likewise, a second, previously unannotated isoform of *P2rx4* was identified, but the Ensembl transcript of this gene was present in the current candidate transcript module. Of note, both transcripts were the predominantly expressed isoforms for their respective genes.

*P2rx4* encodes the P2X purinoceptor 4 receptor, which is a subtype of the purinergic system of ligand-gated ion channels (Köles et al., 2007) that, when activated, exerts excitatory effects in the central nervous system (Lalo et al., 2007). Several studies have demonstrated that alcohol inhibits the excitatory effects of this receptor (Li et al., 2000; Davies et al., 2002; Xiao et al., 2008; Asatryan et al., 2010). Moreover, alcohol preferring P rats show lower functional expression of this gene in brain compared to alcohol nonpreferring NP rats (Kimpel et al., 2007), and alcohol modulates receptor expression (Gofman et al., 2014). Likewise, our previous work found a negative correlation between expression of the *P2rx4* gene as a whole and voluntary alcohol consumption in rats (as observed here) (Tabakoff et al., 2009), and others have likewise corroborated that higher levels of alcohol consumption are associated with lower levels of *P2rx4* gene expression (Kimpel et al., 2007; Tabakoff et al., 2009). In rats treated with alcohol, self-administration was inhibited by a *P2rx4* receptor agonist in rats (Kosten, 2011). These receptors also regulate neuro-inflammatory processes (Gofman et al., 2014). The functional role of *Ift81*—and its potential link with voluntary alcohol consumption—is less clear, although it is a component of cilium formation in astrocytes and neurons (Bhogaraju et al., 2013). Furthermore, cilia, which are neurites, play an important role in brain development including neurogenesis and neuronal migration (Karunakaran et al., 2020).

Beyond *Ift81*, *P2rx4*, and *Lrap*, the other transcripts in the current candidate module include isoforms/transcripts of *Mapkapk5*, *Oas3*, and *AABR07065438.1*.

The *Mapkapk5* (MAPK activated protein kinase 5) transcript in the current candidate module was annotated by StringTie (*MSTRG.6281.1*). MAPK activated protein kinases are enzymes whose activation is mediated by mitogen-activated protein kinases (Cargnello and Roux, 2011). Notably, Mapkapk5 is a downstream target of Mapk14 (New et al., 1998), which was shown to be a central regulator of the immunological response in astrocytes (Lo et al., 2014). *Mapk14* was also differentially expressed in alcohol preferring AA (alko, alcohol) vs alcohol-avoiding (alko, non-alcohol) rats (Arlinde et al., 2004; Sommer et al., 2005). Furthermore, *Mapk14* was expressed at lower levels in alcohol preferring iP rats compared to the alcohol non preferring iNP rats in the caudate-putamen of brain (Rodd et al., 2007). Combining transcripts from the individual transcript analysis and from the current candidate coexpression module, the protein product of *Map3k7*—a candidate individual transcript—is an upstream regulator of Mapk14 (Martín-Blanco, 2000), providing a common biological pathway for *Mapkapk5* and *Map3k7*.

The single transcript for *Oas3*, 2′-5′-oligoadenylate synthetase 3, in the current candidate module was annotated by Ensembl. A genome-wide association study of alcohol consumption in Korean male drinkers identified a SNP in *OAS3* with genome-wide significance (Baik et al., 2011). Similar to Mapkapk5, OAS3 plays a role in immunity, namely the antiviral immune response (Lee et al., 2019, 1). Expression of *Oas3* was shown to be enriched in infiltrating macrophages relative to homeostatic brain microglia during virus-induced neuroinflammation (DePaula-Silva et al., 2019).

The final transcript was derived from the *AABR07065438.1* gene and was identified by StringTie. The single Ensembl annotated transcript for this gene was removed from the DABG transcriptome due to low expression estimates and, therefore, was not included in WGCNA. The Ensembl and StringTie transcripts have identical 5′ and 3′ ends but differ in that the Ensembl transcript has as a single exon, whereas StringTie identified a splice junction and therefore annotated two exons. Ensembl describes the Ensembl transcript as ribosomal protein L6, pseudo 1. Pseudogenes have similar sequence to another gene but are often defective (Vanin, 1985); however, pseudogenes can be transcribed (Kalyana-Sundaram et al., 2012).

Overall, the function of the transcripts in the current candidate module can be linked to inflammation/the immune response. Such an observation is consistent with our previous findings (Saba et al., 2015, Saba et al., 2020).

#### 4.3.2 Candidate Individual Transcripts

Notable candidate transcripts include *Aldh1a7* and *Map3k7*. The individual candidate transcript of *Map3k7* was identified *de novo* and differed from the existing Ensembl transcript for this gene in that exon 12 was skipped (Figure 3). Previous literature has reported that *Map3k7* expresses an exon 12 skipping isoform of the gene (Martinez et al., 2012; Martinez and Lynch, 2013), providing credence for the transcript structure identified here. Specifically, the exon skipping isoform was observed to be differentially expressed in the JSL1 human T-cell line when stimulated to elicit an immune response (Martinez et al., 2012). Regarding alcohol, differences in brain expression of *Map3k7* between high and low alcohol preferring mice have been reported (Hoffman et al., 2014).

The human *ALDH1* family consists of six genes (Black et al., 2009). Aldh1a7 is an additional rodent-specific gene for this family (Touloupi et al., 2019) that is a paralogue of Aldh1a1 (Jackson et al., 2011). Here we annotated a novel transcript for this gene, *ENSRNOT00000024093.1*, which was identified by aptardi. This transcript was present in the DABG transcriptome, while the Ensembl transcript for this gene (*ENSRNOT00000024093*), was removed during filtering. The transcripts shared splice junctions but differ in that the aptardi transcript has a longer 3′ exon. The RNA-Seq reads support the presence of the aptardi transcript (Figure 4). Aldh1a7 is the cytosolic aldehyde dehydrogenase, which metabolizes acetaldehyde and also catalyzes the irreversible conversion of retinaldehyde to retinoic acid (Singh et al., 2013). Retinoic acid can act on immune cells and is involved in neuroinflammation (Oliveira et al., 2018).

These candidate individual transcripts further point towards the role of inflammation and immunity as predisposing factors associated with voluntary alcohol consumption.

### 4.4 Limitations and Future Directions

One limitation of this work is the relatively small sample size for mapping voluntary alcohol consumption (*n* = 21) and for genetic correlations with voluntary alcohol consumption. No individual transcripts were significantly associated with alcohol consumption when correcting for multiple testing (false discovery rate <0.05). However, the fact that inclusion of other data sources enabled the use of multiple, diverse filtering criteria and many of the correlations observed with microarray data were further validated in the RNA-Seq data is evidence for a robust signal.

A second limitation was the ambiguity of the physical location of Map3k7 and whether MSTRG.23809.1 and MSTRG.17584.2 were truly separate transcripts with important difference in the 3′ UTR or if they represent the same transcript. This type of genomic ambiguity often complicates short read RNA-Seq quantitation and further validation of untranslated regions of Map3k7 may be warranted.

Finally, whole brains were used to generate RNA-Seq libraries. Since alternative splicing and alternative polyadenylation display region- and cell-specific expression, whole brain samples may dilute these transcripts to a degree that their abundance is undetectable. However, including entire brains allows for capture of pervasive alternative splicing and alternative polyadenylation in this tissue under our conditions, and future work could complement this study by focusing on particular regions and cell types.

## 5 Conclusion

Current studies on the genetic components of complex traits such as voluntary alcohol consumption often rely on existing annotation. Here we provide a pipeline that enables identification of previously unannotated alternative splicing and alternative polyadenylation transcripts from RNA-Seq data. In the case of the rat brain, we showed that many transcripts are not present in existing annotation and, furthermore, that these previously unknown transcripts may provide important insights into genetic predisposition to voluntary alcohol consumption.

## Data Availability

The alcohol consumption data, the genotype dataset used from mapping alcohol consumption and module eigengenes, the normalized RNA expression levels for the DABG transcriptome, the Gene Transfer File (GTF) for the DABG transcriptome, and the strain-specific genomes for the RI strains that were used from RNA-Seq alignment are on the PhenoGen website [https://phenogen.org/web/sysbio/resources.jsp]. The RNA-Seq data generated and analyzed for this study can be found in the Sequence Read Archive [BioProject ID PRJNA810034]. The single molecule RNA sequencing data is also available through the Sequence Read Archive [BioProject ID: PRJNA801761].

## References

[B1] Abiola O. Angel J. M. Avner P. Bachmanov A. A. Belknap J. K. Bennett B. (2003). The Nature and Identification of Quantitative Trait Loci: a Community's View. Nat. Rev. Genet. 4, 911–916. 10.1038/nrg1206 14634638 PMC2063446

[B2] Arlinde C. Sommer W. Björk K. Reimers M. Hyytiä P. Kiianmaa K. (2004). A Cluster of Differentially Expressed Signal Transduction Genes Identified by Microarray Analysis in a Rat Genetic Model of Alcoholism. Pharmacogenomics J. 4, 208–218. 10.1038/sj.tpj.6500243 15052257

[B3] Asatryan L. Popova M. Perkins D. Trudell J. R. Alkana R. L. Davies D. L. (2010). Ivermectin Antagonizes Ethanol Inhibition in Purinergic P2X4 Receptors. J. Pharmacol. Exp. Ther. 334, 720–728. 10.1124/jpet.110.167908 20543096 PMC2939657

[B4] Baik I. Cho N. H. Kim S. H. Han B.-G. Shin C. (2011). Genome-wide Association Studies Identify Genetic Loci Related to Alcohol Consumption in Korean Men. Am. J. Clin. Nutr. 93, 809–816. 10.3945/ajcn.110.001776 21270382

[B5] Beaudoing E. Gautheret D. (2001). Identification of Alternate Polyadenylation Sites and Analysis of Their Tissue Distribution Using EST Data. Genome Res. 11, 1520–1526. 10.1101/gr.190501 11544195 PMC311108

[B6] Bhogaraju S. Cajanek L. Fort C. Blisnick T. Weber K. Taschner M. (2013). Molecular Basis of Tubulin Transport within the Cilium by IFT74 and IFT81. Science 341, 1009–1012. 10.1126/science.1240985 23990561 PMC4359902

[B7] Black W. J. Stagos D. Marchitti S. A. Nebert D. W. Tipton K. F. Bairoch A. (2009). Human Aldehyde Dehydrogenase Genes: Alternatively Spliced Transcriptional Variants and Their Suggested Nomenclature. Pharmacogenet Genomics 19, 893–902. 10.1097/FPC.0b013e3283329023 19823103 PMC3356695

[B8] Broman K. W. Sen S. (2009). A Guide to QTL Mapping with R/qtl. New York: Springer-Verlag. 10.1007/978-0-387-92125-9

[B9] Bullard J. H. Purdom E. Hansen K. D. Dudoit S. (2010). Evaluation of Statistical Methods for Normalization and Differential Expression in mRNA-Seq Experiments. BMC Bioinformatics 11, 94. 10.1186/1471-2105-11-94 20167110 PMC2838869

[B10] Cargnello M. Roux P. P. (2011). Activation and Function of the MAPKs and Their Substrates, the MAPK-Activated Protein Kinases. Microbiol. Mol. Biol. Rev. 75, 50–83. 10.1128/MMBR.00031-10 21372320 PMC3063353

[B11] Churchill G. A. Doerge R. W. (1994). Empirical Threshold Values for Quantitative Trait Mapping. Genetics 138, 963–971. 10.1093/genetics/138.3.963 7851788 PMC1206241

[B12] Dafne Giammartino D. G. Nishida K. Manley J. L. (2011). Mechanisms and Consequences of Alternative Polyadenylation. Mol. Cel 43, 853–866. 10.1016/j.molcel.2011.08.017 PMC3194005 21925375

[B13] Davies D. L. Machu T. K. Guo Y. Alkana R. L. (2002). Ethanol Sensitivity in ATP-Gated P2X Receptors Is Subunit Dependent. Alcohol. Clin. Exp. Res. 26, 773–778. 10.1111/j.1530-0277.2002.tb02604.x 12068244

[B14] Dawson D. A. Grant B. F. (2011). The "Gray Area" of Consumption between Moderate and Risk Drinking*. J. Stud. Alcohol. Drugs 72, 453–458. 10.15288/jsad.2011.72.453 21513682 PMC3084360

[B15] DePaula-Silva A. B. Gorbea C. Doty D. J. Libbey J. E. Sanchez J. M. S. Hanak T. J. (2019). Differential Transcriptional Profiles Identify Microglial- and Macrophage-specific Gene Markers Expressed during Virus-Induced Neuroinflammation. J. Neuroinflammation 16, 152. 10.1186/s12974-019-1545-x 31325960 PMC6642742

[B16] Derti A. Garrett-Engele P. Macisaac K. D. Stevens R. C. Sriram S. Chen R. (2012). A Quantitative Atlas of Polyadenylation in Five Mammals. Genome Res. 22, 1173–1183. 10.1101/gr.132563.111 22454233 PMC3371698

[B17] Dredge B. K. Polydorides A. D. Darnell R. B. (2001). The Splice of Life: Alternative Splicing and Neurological Disease. Nat. Rev. Neurosci. 2, 43–50. 10.1038/35049061 11253358

[B18] Faustino N. A. Cooper T. A. (2003). Pre-mRNA Splicing and Human Disease. Genes Dev. 17, 419–437. 10.1101/gad.1048803 12600935

[B19] Garcia-Blanco M. A. Baraniak A. P. Lasda E. L. (2004). Alternative Splicing in Disease and Therapy. Nat. Biotechnol. 22, 535–546. 10.1038/nbt964 15122293

[B20] Gibbs R. A. Weinstock G. M. Metzker M. L. Muzny D. M. Sodergren E. J. Scherer S. (2004). Genome Sequence of the Brown Norway Rat Yields Insights into Mammalian Evolution. Nature 428, 493–521. 10.1038/nature02426 15057822

[B21] Gofman L. Cenna J. M. Potula R. (2014). P2X4 Receptor Regulates Alcohol-Induced Responses in Microglia. J. Neuroimmune Pharmacol. 9, 668–678. 10.1007/s11481-014-9559-8 25135400 PMC4209197

[B22] Grant J. D. Agrawal A. Bucholz K. K. Madden P. A. F. Pergadia M. L. Nelson E. C. (2009). Alcohol Consumption Indices of Genetic Risk for Alcohol Dependence. Biol. Psychiatry 66, 795–800. 10.1016/j.biopsych.2009.05.018 19576574 PMC3077105

[B23] Harrall K. K. Kechris K. J. Tabakoff B. Hoffman P. L. Hines L. M. Tsukamoto H. (2016). Uncovering the Liver's Role in Immunity through RNA Co-expression Networks. Mamm. Genome 27, 469–484. 10.1007/s00335-016-9656-5 27401171 PMC5002042

[B24] Hartley C. A. McKenna M. C. Salman R. Holmes A. Casey B. J. Phelps E. A. (2012). Serotonin Transporter Polyadenylation Polymorphism Modulates the Retention of Fear Extinction Memory. Proc. Natl. Acad. Sci. 109, 5493–5498. 10.1073/pnas.1202044109 22431634 PMC3325655

[B25] Havlak P. Chen R. Durbin K. J. Egan A. Ren Y. Song X.-Z. (2004). The Atlas Genome Assembly System. Genome Res. 14, 721–732. 10.1101/gr.2264004 15060016 PMC383319

[B26] Hermsen R. de Ligt J. Spee W. Blokzijl F. Schäfer S. Adami E. (2015). Genomic Landscape of Rat Strain and Substrain Variation. BMC Genomics 16, 357. 10.1186/s12864-015-1594-1 25943489 PMC4422378

[B27] Hoffman P. L. Saba L. M. Flink S. Grahame N. J. Kechris K. Tabakoff B. (2014). Genetics of Gene Expression Characterizes Response to Selective Breeding for Alcohol Preference. Genes, Brain Behav. 13, 743–757. 10.1111/gbb.12175 25160899 PMC4241152

[B28] Hong M. Zhukareva V. Vogelsberg-Ragaglia V. Wszolek Z. Reed L. Miller B. I. (1998). Mutation-specific Functional Impairments in Distinct Tau Isoforms of Hereditary FTDP-17. Science 282, 1914–1917. 10.1126/science.282.5395.1914 9836646

[B29] Hoque M. Ji Z. Zheng D. Luo W. Li W. You B. (2013). Analysis of Alternative Cleavage and Polyadenylation by 3′ Region Extraction and Deep Sequencing. Nat. Methods 10, 133–139. 10.1038/nmeth.2288 23241633 PMC3560312

[B30] Hutton M. Lendon C. L. Rizzu P. Baker M. Froelich S. Houlden H. (1998). Association of Missense and 5′-Splice-Site Mutations in Tau with the Inherited Dementia FTDP-17. Nature 393, 702–705. 10.1038/31508 9641683

[B31] Jackson B. Brocker C. Thompson D. C. Black W. Vasiliou K. Nebert D. W. (2011). Update on the Aldehyde Dehydrogenase Gene (ALDH) Superfamily. Hum. Genomics 5, 283–303. 10.1186/1479-7364-5-4-283 21712190 PMC3392178

[B32] Ji X. Li P. Fuscoe J. C. Chen G. Xiao W. Shi L. (2020). A Comprehensive Rat Transcriptome Built from Large Scale RNA-Seq-Based Annotation. Nucleic Acids Res. 48, 8320–8331. 10.1093/nar/gkaa638 32749457 PMC7470976

[B33] Johnson W. E. Li C. Rabinovic A. (2007). Adjusting Batch Effects in Microarray Expression Data Using Empirical Bayes Methods. Biostatistics 8, 118–127. 10.1093/biostatistics/kxj037 16632515

[B34] Kalyana-Sundaram S. Kumar-Sinha C. Shankar S. Robinson D. R. Wu Y.-M. Cao X. (2012). Expressed Pseudogenes in the Transcriptional Landscape of Human Cancers. Cell 149, 1622–1634. 10.1016/j.cell.2012.04.041 22726445 PMC3597446

[B35] Karunakaran K. B. Chaparala S. Lo C. W. Ganapathiraju M. K. (2020). Cilia Interactome with Predicted Protein-Protein Interactions Reveals Connections to Alzheimer's Disease, Aging and Other Neuropsychiatric Processes. Sci. Rep. 10, 15629. 10.1038/s41598-020-72024-4 32973177 PMC7515907

[B36] Kawasawa Y. I. Mohammad S. Son A. I. Morizono H. Basha A. Salzberg A. C. (2017). Genome-wide Profiling of Differentially Spliced mRNAs in Human Fetal Cortical Tissue Exposed to Alcohol. Alcohol 62, 1–9. 10.1016/j.alcohol.2017.05.001 28755746 PMC7336896

[B37] Kelemen O. Convertini P. Zhang Z. Wen Y. Shen M. Falaleeva M. (2013). Function of Alternative Splicing. Gene 514, 1–30. 10.1016/j.gene.2012.07.083 22909801 PMC5632952

[B38] Kent W. J. (2002). BLAT-the BLAST-like Alignment Tool. Genome Res. 12, 656–664. 10.1101/gr.229202 11932250 PMC187518

[B39] Kent W. J. Sugnet C. W. Furey T. S. Roskin K. M. Pringle T. H. Zahler A. M. (2002). The Human Genome Browser at UCSC. Genome Res. 12, 996–1006. 10.1101/gr.229102 12045153 PMC186604

[B40] Kim D. Langmead B. Salzberg S. L. (2015). HISAT: a Fast Spliced Aligner with Low Memory Requirements. Nat. Methods 12, 357–360. 10.1038/nmeth.3317 25751142 PMC4655817

[B41] Kimpel M. W. Strother W. N. McClintick J. N. Carr L. G. Liang T. Edenberg H. J. (2007). Functional Gene Expression Differences between Inbred Alcohol-Preferring and -Non-Preferring Rats in Five Brain Regions. Alcohol 41, 95–132. 10.1016/j.alcohol.2007.03.003 17517326 PMC1976291

[B42] Kosten T. A. (2011). Pharmacologically Targeting the P2rx4 Gene on Maintenance and Reinstatement of Alcohol Self-Administration in Rats. Pharmacol. Biochem. Behav. 98, 533–538. 10.1016/j.pbb.2011.02.026 21402096 PMC3081972

[B43] Lalo U. Verkhratsky A. Pankratov Y. (2007). Ivermectin Potentiates ATP-Induced Ion Currents in Cortical Neurones: Evidence for Functional Expression of P2X4 Receptors? Neurosci. Lett. 421, 158–162. 10.1016/j.neulet.2007.03.078 17566648

[B44] Lander E. Kruglyak L. (1995). Genetic Dissection of Complex Traits: Guidelines for Interpreting and Reporting Linkage Results. Nat. Genet. 11, 241–247. 10.1038/ng1195-241 7581446

[B45] Langfelder P. Horvath S. (2008). WGCNA: an R Package for Weighted Correlation Network Analysis. BMC Bioinformatics 9, 559. 10.1186/1471-2105-9-559 19114008 PMC2631488

[B46] Langmead B. Salzberg S. L. (2012). Fast Gapped-Read Alignment with Bowtie 2. Nat. Methods 9, 357–359. 10.1038/nmeth.1923 22388286 PMC3322381

[B47] Lau A. G. Irier H. A. Gu J. Tian D. Ku L. Liu G. (2010). Distinct 3'UTRs Differentially Regulate Activity-dependent Translation of Brain-Derived Neurotrophic Factor (BDNF). Proc. Natl. Acad. Sci. 107, 15945–15950. 10.1073/pnas.1002929107 20733072 PMC2936648

[B48] Lee W.-B. Choi W. Y. Lee D.-H. Shim H. Kim-Ha J. Kim Y.-J. (2019). OAS1 and OAS3 Negatively Regulate the Expression of Chemokines and Interferon-Responsive Genes in Human Macrophages. BMB Rep. 52, 133–138. 10.5483/bmbrep.2019.52.2.129 30078389 PMC6443328

[B49] Leek J. T. Johnson W. E. Parker H. S. Jaffe A. E. Storey J. D. (2012). The Sva Package for Removing Batch Effects and Other Unwanted Variation in High-Throughput Experiments. Bioinformatics 28, 882–883. 10.1093/bioinformatics/bts034 22257669 PMC3307112

[B50] Li B. Dewey C. N. (2011). RSEM: Accurate Transcript Quantification from RNA-Seq Data with or without a Reference Genome. BMC Bioinformatics 12, 323. 10.1186/1471-2105-12-323 21816040 PMC3163565

[B51] Li C. Xiong K. Weight F. F. (2000). Ethanol Inhibition of Adenosine 5′-Triphosphate-Activated Current in Freshly Isolated Adult Rat Hippocampal CA1 Neurons. Neurosci. Lett. 295, 77–80. 10.1016/s0304-3940(00)01586-x 11090978

[B52] Li H. Handsaker B. Wysoker A. Fennell T. Ruan J. Homer N. (2009). The Sequence Alignment/Map Format and SAMtools. Bioinformatics 25, 2078–2079. 10.1093/bioinformatics/btp352 19505943 PMC2723002

[B53] Li H. (2018). Minimap2: Pairwise Alignment for Nucleotide Sequences. Bioinformatics 34, 3094–3100. 10.1093/bioinformatics/bty191 29750242 PMC6137996

[B54] L. Koles L. S. Furst S. P. Illes P. (2007). Purine Ionotropic (P2X) Receptors. Cpd 13, 2368–2384. 10.2174/138161207781368747 17692007

[B55] Lo U. Selvaraj V. Plane J. M. Chechneva O. V. Otsu K. Deng W. (2014). p38α (MAPK14) Critically Regulates the Immunological Response and the Production of Specific Cytokines and Chemokines in Astrocytes. Sci. Rep. 4, 7405. 10.1038/srep07405 25502009 PMC4264013

[B56] Love M. I. Huber W. Anders S. (2014). Moderated Estimation of Fold Change and Dispersion for RNA-Seq Data with DESeq2. Genome Biol. 15, 550. 10.1186/s13059-014-0550-8 25516281 PMC4302049

[B57] Lusk R. Saba L. M. Vanderlinden L. A. Zidek V. Silhavy J. Pravenec M. (2018). Unsupervised, Statistically Based Systems Biology Approach for Unraveling the Genetics of Complex Traits: A Demonstration with Ethanol Metabolism. Alcohol. Clin. Exp. Res. 42, 1177–1191. 10.1111/acer.13763 29689131 PMC6028286

[B58] Lusk R. Stene E. Banaei-Kashani F. Tabakoff B. Kechris K. Saba L. M. (2021). Aptardi Predicts Polyadenylation Sites in Sample-specific Transcriptomes Using High-Throughput RNA Sequencing and DNA Sequence. Nat. Commun. 12, 1652. 10.1038/s41467-021-21894-x 33712618 PMC7955126

[B59] MacDonald C. C. (2019). Tissue‐specific Mechanisms of Alternative Polyadenylation: Testis, Brain, and beyond (2018 Update). WIREs RNA 10, e1526. 10.1002/wrna.1526 30816016 PMC6617714

[B60] Manning K. S. Cooper T. A. (2017). The Roles of RNA Processing in Translating Genotype to Phenotype. Nat. Rev. Mol. Cel Biol 18, 102–114. 10.1038/nrm.2016.139 PMC5544131 27847391

[B61] Marioni J. C. Mason C. E. Mane S. M. Stephens M. Gilad Y. (2008). RNA-seq: An Assessment of Technical Reproducibility and Comparison with Gene Expression Arrays. Genome Res. 18, 1509–1517. 10.1101/gr.079558.108 18550803 PMC2527709

[B62] Martin M. (2011). Cutadapt Removes Adapter Sequences from High-Throughput Sequencing Reads. EMBnet j. 17, 10–12. 10.14806/ej.17.1.200

[B63] Martín-Blanco E. (2000). p38 MAPK Signalling Cascades: Ancient Roles and New Functions. Bioessays 22, 637–645. 10.1002/1521-1878(200007)22:7<637::AID-BIES6>3.0.CO;2-E 10878576

[B64] Martinez N. M. Lynch K. W. (2013). Control of Alternative Splicing in Immune Responses: many Regulators, many Predictions, Much Still to Learn. Immunol. Rev. 253, 216–236. 10.1111/imr.12047 23550649 PMC3621013

[B65] Martinez N. M. Pan Q. Cole B. S. Yarosh C. A. Babcock G. A. Heyd F. (2012). Alternative Splicing Networks Regulated by Signaling in Human T Cells. RNA 18, 1029–1040. 10.1261/rna.032243.112 22454538 PMC3334690

[B66] Miura P. Sanfilippo P. Shenker S. Lai E. C. (2014). Alternative Polyadenylation in the Nervous System: To what Lengths Will 3′ UTR Extensions Take Us? Bioessays 36, 766–777. 10.1002/bies.201300174 24903459 PMC4503322

[B67] New L. Jiang Y. Zhao M. Liu K. Zhu W. Flood L. J. (1998). PRAK, a Novel Protein Kinase Regulated by the P38 MAP Kinase. EMBO J. 17, 3372–3384. 10.1093/emboj/17.12.3372 9628874 PMC1170675

[B68] Nilsen T. W. Graveley B. R. (2010). Expansion of the Eukaryotic Proteome by Alternative Splicing. Nature 463, 457–463. 10.1038/nature08909 20110989 PMC3443858

[B69] Nissim-Rafinia M. Kerem B. (2002). Splicing Regulation as a Potential Genetic Modifier. Trends Genet. 18, 123–127. 10.1016/s0168-9525(01)02619-1 11858835

[B70] Oliveira L. d. M. Teixeira F. M. E. Sato M. N. (2018). Impact of Retinoic Acid on Immune Cells and Inflammatory Diseases. Mediators Inflamm. 2018, 1–17. 10.1155/2018/3067126 PMC6109577 30158832

[B71] Pan Q. Shai O. Lee L. J. Frey B. J. Blencowe B. J. (2008). Deep Surveying of Alternative Splicing Complexity in the Human Transcriptome by High-Throughput Sequencing. Nat. Genet. 40, 1413–1415. 10.1038/ng.259 18978789

[B72] Pertea M. Pertea G. M. Antonescu C. M. Chang T.-C. Mendell J. T. Salzberg S. L. (2015). StringTie Enables Improved Reconstruction of a Transcriptome from RNA-Seq Reads. Nat. Biotechnol. 33, 290–295. 10.1038/nbt.3122 25690850 PMC4643835

[B73] Petruccelli E. Brown T. Waterman A. Ledru N. Kaun K. R. (2020). Alcohol Causes Lasting Differential Transcription in Drosophila Mushroom Body Neurons. Genetics 215, 103–116. 10.1534/genetics.120.303101 32132098 PMC7198272

[B74] Pravenec M. Klír P. Kren V. Zicha J. Kunes J. (1989). An Analysis of Spontaneous Hypertension in Spontaneously Hypertensive Rats by Means of New Recombinant Inbred Strains. J. Hypertens. 7, 217–221. 10.1097/00004872-198903000-00008 2708818

[B75] Pravenec M. Saba L. M. Zídek V. Landa V. Mlejnek P. Šilhavý J. (2018). Systems Genetic Analysis of Brown Adipose Tissue Function. Physiol. Genomics 50, 52–66. 10.1152/physiolgenomics.00091.2017 29127223 PMC5866413

[B76] Ren F. Zhang N. Zhang L. Miller E. Pu J. J. (2020). Alternative Polyadenylation: a New Frontier in post Transcriptional Regulation. Biomark Res. 8, 67. 10.1186/s40364-020-00249-6 33292571 PMC7690165

[B77] Rhinn H. Qiang L. Yamashita T. Rhee D. Zolin A. Vanti W. (2012). Alternative α-synuclein Transcript Usage as a Convergent Mechanism in Parkinson's Disease Pathology. Nat. Commun. 3, 1084. 10.1038/ncomms2032 23011138 PMC3660047

[B78] Risso D. Schwartz K. Sherlock G. Dudoit S. (2011). GC-content Normalization for RNA-Seq Data. BMC Bioinformatics 12, 480. 10.1186/1471-2105-12-480 22177264 PMC3315510

[B79] Rodd Z. A. Bertsch B. A. Strother W. N. Le-Niculescu H. Balaraman Y. Hayden E. (2007). Candidate Genes, Pathways and Mechanisms for Alcoholism: an Expanded Convergent Functional Genomics Approach. Pharmacogenomics J. 7, 222–256. 10.1038/sj.tpj.6500420 17033615

[B80] Saba L. M. Flink S. C. Vanderlinden L. A. Israel Y. Tampier L. Colombo G. (2015). The Sequenced Rat Brain Transcriptome - its Use in Identifying Networks Predisposing Alcohol Consumption. Febs J. 282, 3556–3578. 10.1111/febs.13358 26183165 PMC4573833

[B81] Saba L. M. Hoffman P. L. Homanics G. E. Mahaffey S. Daulatabad S. V. Janga S. C. (2020). A Long Non‐coding RNA ( Lrap ) Modulates Brain Gene Expression and Levels of Alcohol Consumption in Rats. Genes. Brain Behav. 20, e12698. 10.1111/gbb.12698 32893479 PMC7900948

[B82] Sanfilippo P. Wen J. Lai E. C. (2017). Landscape and Evolution of Tissue-specific Alternative Polyadenylation across Drosophila Species. Genome Biol. 18, 229. 10.1186/s13059-017-1358-0 29191225 PMC5707805

[B83] Sasabe T. Ishiura S. (2010). Alcoholism and Alternative Splicing of Candidate Genes. Ijerph 7, 1448–1466. 10.3390/ijerph7041448 20617039 PMC2872348

[B84] Sen Ś. Churchill G. A. (2001). A Statistical Framework for Quantitative Trait Mapping. Genetics 159, 371–387. 10.1093/genetics/159.1.371 11560912 PMC1461799

[B85] Shannon P. Markiel A. Ozier O. Baliga N. S. Wang J. T. Ramage D. (2003). Cytoscape: A Software Environment for Integrated Models of Biomolecular Interaction Networks. Genome Res. 13, 2498–2504. 10.1101/gr.1239303 14597658 PMC403769

[B86] Singh S. Brocker C. Koppaka V. Chen Y. Jackson B. C. Matsumoto A. (2013). Aldehyde Dehydrogenases in Cellular Responses to Oxidative/electrophilicstress. Free Radic. Biol. Med. 56, 89–101. 10.1016/j.freeradbiomed.2012.11.010 23195683 PMC3631350

[B87] Smit A. Hubley R. Green P. (1996). RepeatMasker‬ Open-3.01996. Available at: http://www.repeatmasker.org (Accessed November‬‬‬‬‬ 22, 2018).

[B88] Sommer W. Arlinde C. Heilig M. (2005). The Search for Candidate Genes of Alcoholism: Evidence from Expression Profiling Studies. Addict. Biol. 10, 71–79. 10.1080/13556210412331327821 15849021

[B89] Spillantini M. G. Murrell J. R. Goedert M. Farlow M. R. Klug A. Ghetti B. (1998). Mutation in the Tau Gene in Familial Multiple System Tauopathy with Presenile Dementia. Proc. Natl. Acad. Sci. 95, 7737–7741. 10.1073/pnas.95.13.7737 9636220 PMC22742

[B90] STAR Consortium (2008). SNP and Haplotype Mapping for Genetic Analysis in the Rat. Nat. Genet. 40, 560–566. 10.1038/ng.124 18443594 PMC5915293

[B91] Tabakoff B. Saba L. Saba L. Printz M. Flodman P. Hodgkinson C. (2009). Genetical Genomic Determinants of Alcohol Consumption in Rats and Humans. BMC Biol. 7, 70. 10.1186/1741-7007-7-70 19874574 PMC2777866

[B92] Tabakoff B. Smith H. Vanderlinden L. A. Hoffman P. L. Saba L. M. (2019). Networking in Biology: The Hybrid Rat Diversity Panel. Methods Mol. Biol. 2018, 213–231. 10.1007/978-1-4939-9581-3_10 31228159

[B93] Tian B. Manley J. L. (2017). Alternative Polyadenylation of mRNA Precursors. Nat. Rev. Mol. Cel Biol 18, 18–30. 10.1038/nrm.2016.116 PMC5483950 27677860

[B94] Touloupi K. Küblbeck J. Magklara A. Molnár F. Reinisalo M. Konstandi M. (2019). The Basis for Strain-dependent Rat Aldehyde Dehydrogenase 1A7 (ALDH1A7) Gene Expression. Mol. Pharmacol. 96, 655–663. 10.1124/mol.119.117424 31575620

[B95] Van Booven D. Mengying Linull, Sunil Rao J. Sunil Rao J. Blokhin I. O. Dayne Mayfield R. Barbier E. (2021). Alcohol Use Disorder Causes Global Changes in Splicing in the Human Brain. Transl Psychiatry 11, 2. 10.1038/s41398-020-01163-z 33414398 PMC7790816

[B96] Vanderlinden L. A. Saba L. M. Printz M. P. Flodman P. Koob G. Richardson H. N. (2014). Is the Alcohol Deprivation Effect Genetically Mediated? Studies with HXB/BXH Recombinant Inbred Rat Strains. Alcohol. Clin. Exp. Res. 38, 2148–2157. 10.1111/acer.12471 24961585 PMC4142977

[B97] Vanin E. F. (1985). Processed Pseudogenes: Characteristics and Evolution. Annu. Rev. Genet. 19, 253–272. 10.1146/annurev.ge.19.120185.001345 3909943

[B98] Verhulst B. Neale M. C. Kendler K. S. (2015). The Heritability of Alcohol Use Disorders: a Meta-Analysis of Twin and Adoption Studies. Psychol. Med. 45, 1061–1072. 10.1017/S0033291714002165 25171596 PMC4345133

[B99] Wang E. T. Sandberg R. Luo S. Khrebtukova I. Zhang L. Mayr C. (2008). Alternative Isoform Regulation in Human Tissue Transcriptomes. Nature 456, 470–476. 10.1038/nature07509 18978772 PMC2593745

[B100] Wang G.-S. Cooper T. A. (2007). Splicing in Disease: Disruption of the Splicing Code and the Decoding Machinery. Nat. Rev. Genet. 8, 749–761. 10.1038/nrg2164 17726481

[B101] Winkler A. Mahal B. Kiianmaa K. Zieglgänsberger W. Spanagel R. (1999). Effects of Chronic Alcohol Consumption on the Expression of Different NR1 Splice Variants in the Brain of AA and ANA Lines of Rats. Mol. Brain Res. 72, 166–175. 10.1016/s0169-328x(99)00218-1 10529475

[B102] Xiao C. Zhou C. Li K. Davies D. L. Ye J. H. (2008). Purinergic Type 2 Receptors at GABAergic Synapses on Ventral Tegmental Area Dopamine Neurons Are Targets for Ethanol Action. J. Pharmacol. Exp. Ther. 327, 196–205. 10.1124/jpet.108.139766 18583548 PMC2861430

[B103] Yates A. D. Achuthan P. Akanni W. Allen J. Allen J. Alvarez-Jarreta J. (2020). Ensembl 2020. Nucleic Acids Res. 48, D682–D688. 10.1093/nar/gkz966 31691826 PMC7145704

[B104] Yeh H.-S. Yong J. (2016). Alternative Polyadenylation of mRNAs: 3′-Untranslated Region Matters in Gene Expression. Mol. Cell 39, 281–285. 10.14348/molcells.2016.0035 PMC4844933 26912084

[B105] Yoon O. K. Hsu T. Y. Im J. H. Brem R. B. (2012). Genetics and Regulatory Impact of Alternative Polyadenylation in Human B-Lymphoblastoid Cells. Plos Genet. 8, e1002882. 10.1371/journal.pgen.1002882 22916029 PMC3420953

[B106] Yoon Y. McKenna M. C. Rollins D. A. Song M. Nuriel T. Gross S. S. (2013). Anxiety-associated Alternative Polyadenylation of the Serotonin Transporter mRNA Confers Translational Regulation by hnRNPK. Proc. Natl. Acad. Sci. 110, 11624–11629. 10.1073/pnas.1301485110 23798440 PMC3710847

[B107] Zhang B. Horvath S. (2005). A General Framework for Weighted Gene Co-expression Network Analysis. Stat. Appl. Genet. Mol. Biol. 4, Article17. 10.2202/1544-6115.1128 16646834

[B108] Zhang H. Lee J. Tian B. (2005). Biased Alternative Polyadenylation in Human Tissues. Genome Biol. 6, R100. 10.1186/gb-2005-6-12-r100 16356263 PMC1414089

