# Supplementary material for: Beyond Genes: Inclusion of Alternative Splicing and Alternative Polyadenylation to Assess the Genetic Architecture of Predisposition to Voluntary Alcohol Consumption in Brain of the HXB/BXH Recombinant Inbred Rat Panel
Source: Front Genet. 2022 Mar 15;13:821026. doi: 10.3389/fgene.2022.821026 (PMC8965255; doi:10.3389/fgene.2022.821026)
Supplement: Supplementary file 1 [file DataSheet3.docx]

Supplementary Material

# Supplementary Figures and Tables

## Supplementary Figures

**Supplementary Figure 1. A general overview of the steps used to generate the detected above background (DABG) transcriptome.** Starting with the Ensembl reference transcriptome, StringTie incorporated short-read RNA sequencing (RNA-Seq) data to identify expressed transcripts. aptardi likewise utilized RNA-Seq, as well as DNA sequence, to identify transcripts with different 3’ termini than the input StringTie transcriptome. Finally, transcripts with low RNA expression levels were removed by quantitating with RSEM and then identifying transcripts with zero counts in one third or more of samples or 200 bases or fewer in length. This was followed by re-quantitation and once again removal of transcripts with zero counts in one third or more of samples. Yellow boxes indicate transcriptomes, and blue boxes indicate data used during transcript assembly.

**Supplementary Figure 2. A detailed flowchart of the steps used to generate and quantitate the detected above background (DABG) transcriptome.**

**Supplementary Figure 3. Outline of the steps used to identify candidate coexpression modules and transcripts.** Only the top three expressed isoforms per gene using the mean transcript per million value across individual rat RNA sequencing samples for rats with alcohol consumption data only (63 samples, 21 strains) were considered for association with alcohol consumption and weighted gene coexpression network analysis (WGCNA). Next, only transcripts with high heritability (> 0.478) were included to focus on genetically influenced transcripts. Finally, transcripts that could be associated with a gene symbol through Ensembl were included for interpretability. (These steps are denoted by yellow boxes.) Using these transcripts, candidate coexpression modules were identified using WGCNA and requiring 1) module eigengene association with voluntary alcohol consumption (Spearman’s rank correlation coefficient p-value <= 0.01) and 2) a significant module eigengene QTL (genome-wide p-value < 0.01) overlap with voluntary alcohol consumption QTL (genome-wide p-value < 0.63) using 95% Bayesian credible intervals (green boxes). Individual candidate transcripts were identified by requiring 1) expression correlation with voluntary alcohol consumption (Spearman’s rank correlation coefficient p-value <= 0.01) and 2) a significant expression QTL (genome-wide p-value < 0.01) overlap with voluntary alcohol consumption QTL (genome-wide p-value < 0.63) using 95% Bayesian credible intervals (orange boxes). Strain means of the normalized expression values were used as expression estimates for the transcripts in WGCNA/individual transcript associations, and strain mean voluntary alcohol consumption values were used for the association analyses. Heritability was estimated as the coefficient of determination (R^2^) value from a one-way ANOVA using strain as the predictor (30 strains total) and transcript normalized expression estimates from individual rats as the response. Each strain possessed three expression estimates per transcript.

B

A

**Supplementary Figure 4. Network topology as a function of soft-thresholding power (β).** The influence of different index values on the goodness-of-fit to scale-free topology and topological features for the network were examined in **(A)** scale-free model fit index vs β and **(B)** mean connectivity vs β. Each point is labeled by its β index value in red. An index of seven was chosen for network construction.

**Supplementary Figure 5. Number of isoforms for each gene in (A) the reference transcriptome and (B) the detection above background transcriptome.** Numbers above each bar in red indicate the percentage of total genes.

**Supplementary Figure 6. Voluntary alcohol consumption quantitative trait loci (QTL). A) Original QTL for alcohol consumption.** The red plot represents the original QTL for alcohol consumption in the HXB/BXH recombinant inbred rat panel. **B) QTL adjusted for the maximum peak on chromosome 1.** The green plot represents the QTL scan for alcohol consumption after adjusting for the maximum peak chromosome 1 QTL (chr1:239 Mb). The green line displays the logarithm of odds (LOD) suggestive threshold (p-value < 0.63) for genome-wide significance in the original analysis. The LOD scores below the suggestive threshold after adjusting for the maximum peak chromosome 1 QTL suggest that the two adjacent peaks on chromosome 1 display some degree of linkage disequilibrium and are better represented by a single QTL. The peaks on chromosome 5 and chromosome 12 remain suggestive after the adjustment, indicating that these two peaks contribute independently of chromosome 1 to the phenotype of voluntary alcohol consumption.

**Supplementary Figure 7. Distribution of module sizes built from weighted gene coexpression network analysis of the brain RNA expression data in the HXB/BXH recombinant inbred rat panel.** Module size is defined as the number of transcripts in the module. Modules with 30 or more transcripts were put into a single bin (30+). The red number on top of each bar indicates the proportion of all modules have that number of transcripts.

**Supplementary Figure 8. Module memberships of transcripts derived from genes expressing more than one isoform and included in weighted gene coexpression network analysis (WGCNA).** The y-axis indicates the number of genes with more than one transcript (i.e., isoform). On the x-axis genes have been separated into two groups based on the total number of isoforms of the gene included in WGCNA. Blue bars indicate genes whose isoforms all belong to the same coexpression module, and red bars indicate genes whose isoforms belong to multiple modules.

**Supplementary Figure 9. Isoforms of the *Mapkapk5* gene.** Blue transcripts represent those identified in the detection above background (DABG) transcriptome, the red transcripts represent the transcripts present in Ensembl reference annotation, and the green transcripts represent the transcripts identified through single molecule RNA sequencing in brain of F344/Stm and LE/Stm strains. *ENSRNOT00000001817* and *ENSRNOT00000065314* are annotated in the reference transcriptome and retained in the DABG transcriptome, whereas *MSTRG.6281.1* represents a novel isoform identified here by StringTie. The RNA sequencing reads on the positive strand (black plot) represent a 10% randomly sampled subset from the HXB/BXH recombinant inbred rat panel RNA sequencing data in brain. This image was generated using the UCSC Genome Browser (<http://genome.ucsc.edu>).

## Supplementary Tables

**Supplementary Table 1. The number of paired end reads from each HXB/BXH recombinant inbred rats strain RNA sequencing library after processing reads for quality.**

**Supplementary Table 2. The genome alignment rate for each HXB/BXH recombinant inbred rat strain RNA sequencing library after processing for quality.** Libraries were aligned to strain specific genomes using HISAT2.

**Supplementary Table 3. Unique 3’ termini identified by aptardi already annotated by a StringTie or reference transcript 3’ terminus (+/- 100 bases) before filtering (aptardi) and after filtering to generate the detection above background (DABG) transcriptome.**

**Supplementary Table 4. Overview of the strains with data available and the number of strains used in each analysis.** The upper portion of the table includes the data types and strains possessing the data. The lower portion of the table includes the analysis type and datasets used for analysis. Only strains with data for each data type required in the analysis were included.
